# The effect of EMS, IFC, and TENS on patient-reported outcome measures for chronic low back pain: a systematic review and meta-analysis

**DOI:** 10.3389/fpain.2024.1346694

**Published:** 2024-06-24

**Authors:** Daniel Wolfe, Brent Rosenstein, Maryse Fortin

**Affiliations:** Department of Health, Kinesiology, and Applied Physiology, Concordia University, Montreal, QC, Canada

**Keywords:** TENS, EMS, NMES, CLBP, chronic low back pain, IFC, pain

## Abstract

**Introduction:**

Chronic low back pain (CLBP) is the leading cause of years lived with disability worldwide. Transcutaneous electrotherapies have been widely used to treat CLBP but, with the partial exception of transcutaneous electrical nerve stimulation (TENS), their effect on pain, disability, quality-of-life, and psychosocial outcomes have not been systematically reviewed. The purpose of this systematic review and meta-analysis was to clarify the overall effect of transcutaneous electrotherapies on patient-reported outcome measures (PROMs) in CLBP patients.

**Methods:**

Four databases and two study registries were searched for studies that utilized transcutaneous electrotherapies as a primary intervention for CLBP, compared against active or passive controls. Two reviewers independently extracted study data and assessed risk of bias. Studies were grouped by intervention vs. comparison, and by time of follow-up. Meta-analyses were conducted where appropriate.

**Results:**

A total of 89 full-text were assessed for eligibility; 14 studies were included, with 6 in the meta-analyses (all TENS or mixed TENS). Pain: meta-analyses revealed no significant difference for TENS vs. active control, TENS vs. passive control, or mixed TENS vs. active control at post-intervention, nor for mixed TENS vs. active control at 1-month post-intervention. Interferential current (IFC) was more effective than active control (2 studies), while electromyostimulation (EMS) was generally superior to passive, but not active, controls (6 studies).

**Disability:**

Meta-analyses revealed no significant difference for TENS vs. active control at post-intervention, mixed TENS vs. active control at post-intervention, or mixed TENS vs. active control at 1-month post-intervention. IFC was more effective than active control (2 studies), while the EMS results were mixed (6 studies). We were unable to perform meta-analyses for quality-of-life or psychosocial outcomes.

**Conclusion:**

There is *moderate* evidence that TENS is similar to all controls for improving pain and disability. There is *limited* evidence that IFC is superior to active controls for improving pain and disability. There is *limited* evidence that EMS is superior to passive but not active controls for improving pain, and similar to all controls for improving disability.

**Systematic Review Registration:**

https://www.crd.york.ac.uk/prospero/display_record.php?RecordID=452851, Identifier (CRD42023452851).

## 1 Introduction

Chronic low back pain (CLBP) affects approximately 20% of the global population (1) and is the leading cause of years-lived with disability worldwide (2), in spite of large healthcare expenditures towards its treatment (3). CLBP is a multi-factorial condition (4–6) and psychological variables (e.g., pain catastrophizing, fear-avoidance beliefs) can mediate CLBP-related patient prognosis (5, 6). To date, however, guidelines for the development of systematic reviews for CLBP interventions have not recommended examining fear-avoidance beliefs, fear of movement, and pain catastrophizing as part of patient-centered outcomes (7, 8).

Transcutaneous electrotherapies are routine conservative interventions for CLBP, typically used for analgesic (e.g., transcutaneous electrical nerve stimulation, interferential current) or motor stimulation (e.g., neuromuscular electrical nerve stimulation, Russian current) purposes. Transcutaneous electrical nerve stimulation (TENS) aims to stimulate sensory nerve fibres, and is thought to promote analgesia by activating endogenous inhibitory pathways in the central nervous system and by reducing peripheral nociceptive output (9). Neuromuscular electrical nerve stimulation (NMES) is a form of electromyostimulation (EMS) that evokes visible muscle contractions (10–12) in order to improve muscle activation and strength (10). While NMES is traditionally delivered under static conditions (10, 11) or superimposed with voluntary isometric contractions (11), whole-body electromyostimulation (WB-EMS) is a recent iteration of NMES which delivers current through a form-fitted suit and allows for whole-body resistance training in conjunction with muscle stimulation (13) Handheld TENS and NMES units deliver current at frequencies that typically range from 2 to 300 Hz (10). In contrast, larger, medium-frequency, stand-alone units (14–16) can produce carrier frequencies ranging from 1 to 4 kHz, allowing for a variety of clinical applications (10, 14, 15, 17). For example, interferential current (IFC) is a medium-frequency treatment typically used for pain relief (10, 17, 18), while Russian (10, 12) and Aussie (14) current are medium-frequency iterations of EMS. Proponents of medium-frequency electrostimulation argue that it lowers skin impedance (19), allowing current to penetrate tissue more deeply than low-frequency alternatives, such as TENS and NMES, and providing a more comfortable experience for users (10, 12).

To date, although a wide spectrum of randomized controlled trials have investigated transcutaneous electrotherapies for CLBP, systematic reviews have overwhelmingly focused on the efficacy of TENS (20–26). Recommendations remain equivocal: some medical guidelines do not recommend TENS for CLBP (27, 28), while recent systematic reviews suggest it improves pain (25) and disability (26) under certain conditions. However, little information is known about the effect of other transcutaneous electrotherapies on PROMs for CLBP. A single meta-analysis that was not CLBP-specific examined the effect of IFC on pain in musculoskeletal conditions (17), and no systematic reviews have been able to evaluate the effect of EMS on PROMs in CLBP patients. The Philadelphia Panel (2001) was unable to find eligible studies investigating the effect of EMS in subjects with CLBP (29). Poitras and Brosseau (2008) attempted to assess the effect of EMS in CLBP, as part of a larger review article, but found no relevant studies (24). The lack of data regarding the efficacy of electrotherapies aside from TENS is clinically relevant: medium-frequency interventions may be more comfortable for patients than low-frequency interventions (10), and recent systematic reviews examining the effect of EMS on paraspinal muscle characteristics have reported that EMS improves paraspinal muscle strength (30, 31) and endurance (30, 31) in CLBP patients. Considered collectively, non-TENS electrotherapies might offer a compelling and more holistic alternative to TENS if they help improve patients' self-reported symptoms, but this remains to be determined.

Therefore, the aim of this systematic review was to assess the overall effect of transcutaneous electrotherapy (e.g., EMS, IFC and TENS) on PROMs for CLBP including pain, back-related disability, fear-avoidance behaviours, catastrophizing beliefs, and quality of life. Focus was paid to short (<3 months), medium (3–12 months), and long-term (>12 months) outcomes.

## 2 Methods

This systematic review protocol was registered with PROSPERO (ID number: CRD42023452851). In designing the protocol, we followed the recommendations suggested by the Preferred Reporting Items for Systematic reviews and Meta-Analyses (PRISMA) 2020 explanation and elaboration guidebook (32). We also adhered to the guidelines proposed by Cochrane Neck and Back (33) for systematic reviews.

### 2.1 Selection criteria

#### 2.1.1 Types of studies

We included randomized and quasi-randomized control trials assessing the effect of transcutaneous electrotherapy in CLBP patients in comparison with a passive or active control (defined below). In line with the Philadelphia Panel's consensus opinion (29), only studies with ≥5 participants per treatment group were included. To adequately assess the effect of interventions over time, we only included studies with ≥8 treatments per group. Only English or French language articles were included.

#### 2.1.2 Participants

Participants aged 18–70 with a diagnosis of CLBP, defined as persistent pain between the lower ribs and gluteal fold, with or without leg pain, of at least 12 weeks duration were included in this review. Participants diagnosed with a specific spinal pathology, defined as one of the following: infection, tumour, previous lumbar surgery, osteoporosis, fracture, structural deformity (ex. scoliosis), inflammatory disorder (ex. ankylosis spondylosis), or cauda equina syndrome, were excluded. However, participants with lumbar disc herniation were included—provided they did not present with radicular symptoms—in line with evidence that disc degeneration and annulus tears (visible on T2-weighted MRI) are not necessarily painful (34). Studies that included participants with mixed lower and upper back pain were excluded. Additionally, studies that included a mix of acute (<4 weeks) and chronic LBP patients, as well as sub-acute (4–12 weeks) and chronic LBP patients were excluded, in line with suggestions that these conditions be considered separately (35).

#### 2.1.3 Types of interventions

We included studies that use transcutaneous electrotherapy as the primary intervention for CLBP. In cases of studies using transcutaneous electrotherapy *plus* another intervention, transcutaneous electrotherapy had to account for at least 40% of the treatment program. This cut-off value was previously used by Macedo et al. (2009) in their systematic review of motor control exercises for persistent LBP (35). Studies that compared the effect of transcutaneous electrotherapy against a passive or active control were included. Passive controls were defined as the following: sham electrotherapy (defined as having the device modified so that no current passes to skin-surface electrodes), usual care, and/or no treatment. Actives controls were defined as the following: any non-transcutaneous electrotherapeutic intervention for CLBP, such as ultrasound, hot/cold packs, exercise, mobilization/manipulation, massage/soft tissue therapy, acupuncture, and non-transcutaneous electrotherapy. Studies comparing two transcutaneous electrotherapeutic treatments were be excluded, as were studies comparing the effect of transcutaneous electrotherapy *plus* another intervention against a third intervention (ex. TENS + hot pack vs. ultrasound), in line with recommendations by the Cochrane Back and Neck Group (33) Additionally, studies where the method of determining stimulation intensity is according to manufacturers' specifications, or where it is not described, were excluded.

#### 2.1.4 Types of outcome measures

We included studies that assessed at least one of the following primary outcomes: pain (ex. VAS, NPRS), back-related disability (ex. Oswestry Disability Index, Roland-Morris disability questionnaire), fear-avoidance behaviours (ex. Fear Avoidance Belief Questionnaire), pain catastrophizing (ex. Pain Catastrophizing Scale), quality of life (ex. Short Health Form Survey), patient satisfaction (ex. Patient Satisfaction Survey), and depression (ex. PHQ-9).

### 2.2 Search strategy

The following bibliographic databases were searched for studies pertaining to CLBP and transcutaneous electrotherapy: PubMed, Scopus, Web of Science, and Embase. Additionally, the follow study registers were searched for protocols of the included studies: WHO International Clinical Trials Registry Platform^1^ and the US National Institute of Health.^2^ A search strategy was developed based on a literature review, and with help from a reference librarian at Concordia University affiliated with the department of Health, Kinesiology, and Applied Physiology. Mesh terms and key words related to: (1) Low back pain, (2) electrical stimulation therapy, (3) TENS, (4) NMES were used. The search strategy for PubMed and Embase is available in Appendix 1. The initial search was performed between February 1, 2022 and March 31, 2022, and the database was updated for the last time on September 15, 2022. No time limit was applied to publication dates. Search results were compiled in the reference management software Zotero (version 5.0.96.3).

### 2.3 Study selection

Two reviewers (DW, BR) initially screened the search results for potential studies based on the study title and, where reasonable, the abstract. After excluded articles during this first round, the full text of the remaining articles was read by the same reviewers, and a global yes/no decision was made for each potential study based on the inclusion criteria identified in Section 2.1. In the case of disagreement over the inclusion of an article, a third reviewer (MF) was consulted, and a consensus decision between the 3 reviewers was taken. Study screening was managed using SR Accelerator.^3^

### 2.4 Data extraction and risk of bias

Two reviewers (DW, BR) independently extracted data from each included study using a modified version of the extraction template developed by Cochrane Back and Neck (33). Participant characteristics, interventions, comparisons, outcomes, analysis approach, results, and study sponsorship were recorded.

Risk of bias was assessed using the revised Cochrane risk-of-bias tool (RoB 2) (36) for randomized trials. This tool examines five domains: randomization bias, bias due to deviations from intended interventions, missing outcome data bias, measurement bias, and bias in selection of the reported result. Bias is assessed on a per-outcome basis, and the tool uses signaling questions and an algorithm to guide reviewers to judgment. Each outcome is rated as follows: low risk, some concerns, high risk. Both reviewers (DW, BR) strictly followed the instructions outlined in the full guidance document for the RoB 2 tool. In case of disagreement, a third reviewer (MF) was consulted.

### 2.5 Statistical analysis

Studies were grouped according to intervention vs. comparator (active or passive), at short, medium, and long term follow up (subgroup analysis). Since not all studies provided change scores, we based our between-group analyses on post-intervention or equivalent (e.g., 1-month post-intervention) scores. Outcome values were pooled where appropriate using StatstoDo^4^. Meta-analyses were conducted, using a random-effects model, when comparisons within a group were sufficiently homogenous with respect to PICO variables (population, intervention, comparator, outcome). A minimum of three comparisons were needed for a comparison to be eligible for meta-analysis; in such cases, the overall treatment effect of the intervention, with 95% confidence intervals, was calculated for each outcome. For continuous variables measured using different scales, the standardized mean difference (SMD) was calculated. Statistical heterogeneity was conducted using the Q-test, and reported as the *I*^2^ statistic. We interpreted the statistic as follows: <40% suggests a low risk of heterogeneity, 40%–75% a moderate heterogeneity, >75% a high risk of heterogeneity (33). We used the Review Manager statistical software (RevMan version 5.4.1) to conduct the meta-analyses.

## 3 Results

### 3.1 Search results

**Figure 1:**
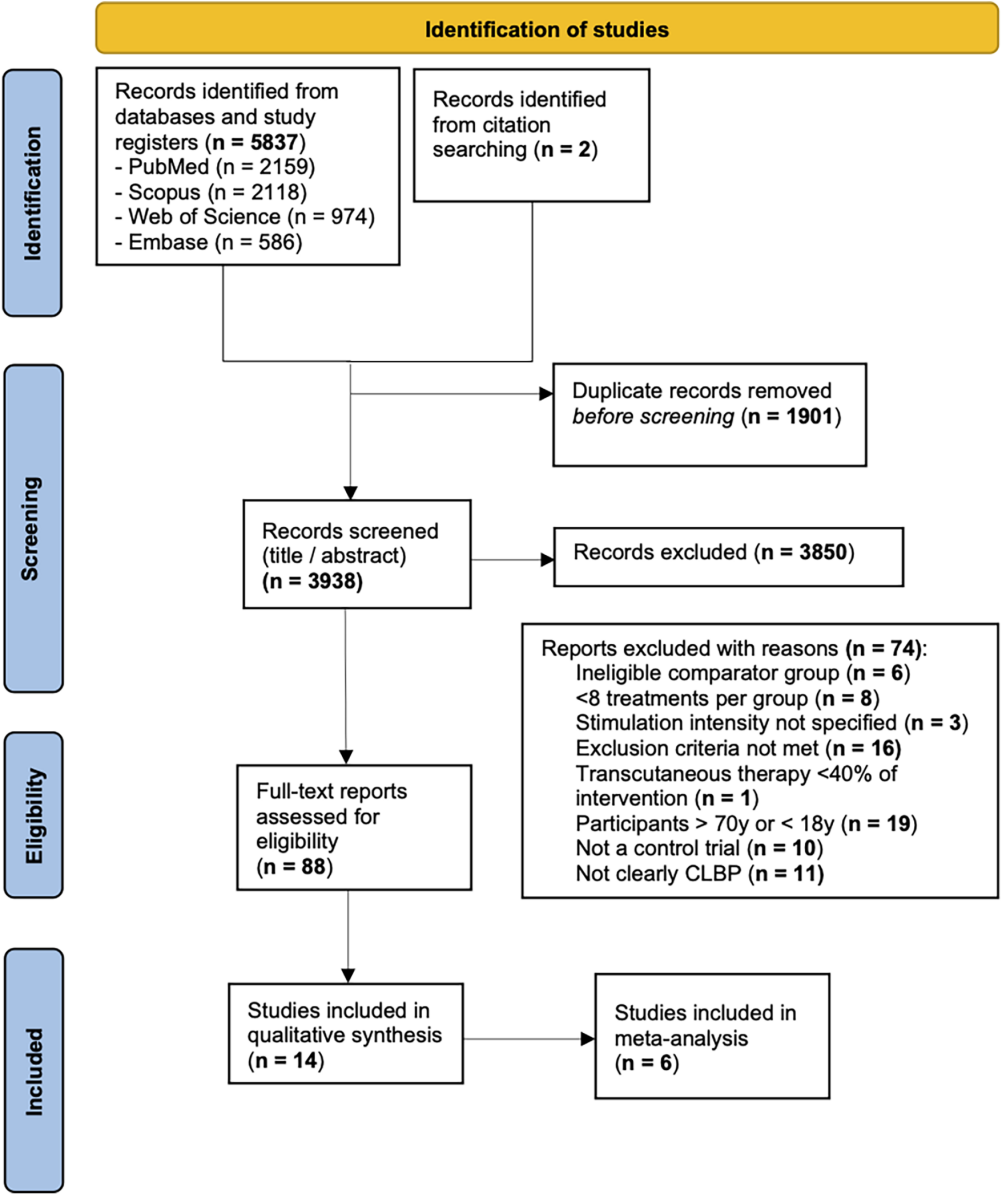
Study flow chart.

We performed an electronic search for eligible articles across the following four databases: PubMed, Embase, Scopus, and Web of Science. We also hand searched reference lists for articles that the electronic search might have missed. A total of 5,839 records were found through the search. After removing duplicates, 3,938 titles were screened and 88 were selected for a full-text review. Fourteen studies were included in the qualitative review and six were included in the meta-analysis. The full results of our search are presented in Figure 1.

### 3.2 Risk of bias

Risk of bias was evaluated on a per-outcome basis and ranged from “some concerns” to “high”. Specifically, *bias in selected of the reported result* was judged to be of at least “some concerns” for all outcomes, because no protocols for statistical analysis could be found for any of the included studies, and therefore a comparison between the published report and the protocol could not be performed, which automatically elevates the risk of bias for this domain. The risk-of-bias assessment is presented in Table 1.

**Table 1:** Risk of bias.

Included study	Outcome variable	Comparison	Bias from the randomization process	Bias due to deviations from intended interventions	Bias due to missing outcome data	Bias in measurement of the outcome	Bias in selection of the reported result	Overall judgment
Albornoz-Cabello et al. (37)	Self-reported	IFC vs. usual care (active control)	Low	Low	Low	Some concerns	Some concerns	Some concerns
Alrwaily et al. (38)	Self-reported	NMES + exercise (mixed) vs. exercise (active control)	Low	Low	Low	Some concerns	High	High
	Fear-avoidance beliefs	NMES + exercise (mixed) vs. exercise (active control)	Low	Low	Low	Some concerns	Some concerns	Some concerns
Batistella et al. (15)	Self-reported	Russian current vs. passive control	Low	Low	Low	High	High	High
Caldas et al. (39)	Self-reported	TENS vs. cryotherapy (active control)	Low	High	High	Some concerns	Some concerns	High
	Self-reported	TENS vs. placebo TENS (passive control)	Low	High	High	Low	Some concerns	High
	Self-reported	TENS + cryotherapy (mixed) vs. cryotherapy (active control)	Low	High	High	Some concerns	Some concerns	High
	Self-reported	TENS + cryotherapy (mixed) vs. placebo TENS (passive control)	Low	High	High	Some concerns	Some concerns	High
Depaoli-Lemos et al. (40)	Self-reported	TENS + exercise (mixed) vs. exercise (active control)	Low	Low	Low	Some concerns	High	High
Dimer daLuz et al. (41)	Self-reported	NMES vs. exercise (active control)	Low	Low	Low	Some concerns	Some concerns	Some concerns
	Self-reported	NMES + exercise (mixed) vs. exercise (active control)	Low	Low	Low	Some concerns	Some concerns	Some concerns
Elserty et al. (42)	Self-reported	TENS vs. exercise (active control)	Low	High	High	Some concerns	Some concerns	High
Kofotolis et al. (43)	Self-reported	TENS vs. exercise (active control)	Some concerns	Low	Low	Some concerns	Some concerns	Some concerns
	Self-reported	TENS vs. placebo TENS (passive control)	Some concerns	Low	Low	Some concerns	Some concerns	Some concerns
	Self-reported	TENS + exercise (mixed) vs. exercise (active control)	Some concerns	Low	Low	Some concerns	Some concerns	Some concerns
	Self-reported	TENS + exercise (mixed) vs. placebo TENS (passive control)	Some concerns	Low	Low	Some concerns	Some concerns	Some concerns
LaraPalomo et al. (16)	Self-reported	IFC vs. superficial massage (active control)	Low	Low	Low	Some concerns	Some concerns	Some concerns
Pelegrini et al. (14)	Self-reported	Aussie Current vs. passive control	Some concerns	Low	Low	High	Some concerns	High
Topuz et al. (44)	Self-reported	TENS vs. placebo TENS (passive control)	Low	High	High	Low	Some concerns	High
	Self-reported	TENS vs. PNT (active control)	Low	High	High	Some concerns	Some concerns	High
Weissenfels et al. (13)	Self-reported	WB-EMS vs. Passive control	Low	Low	Low	High	Some concerns	High
Weissenfels et al. (45)	Self-reported	WB-EMS vs. Exercise (active control)	Low	Low	Low	Low	Some concerns	Some concerns
Yaksi et al. (46)	Self-reported	TENS vs. placebo TENS (passive control)	Some concerns	Low	Low	Some concerns	Some concerns	Some concerns

### 3.3 Characteristics of included studies

Of the fourteen included studies, twelve were stand-alone, and two formed a pair with a shared recruitment process and inclusion/exclusion criteria, but different interventions. Eight studies evaluated sensory electrotherapy: six used TENS (39, 40, 42–44, 46), and two used IFC (16, 37) The remaining six studies evaluated EMS: one used NMES (38), one used Russian current (15), one used Aussie current (14), one used mid-frequency (2,500 Hz) current with progressive low-frequency (LF) modulation (41), and two used WB-EMS (13, 45) Furthermore, eight studies were comprised of stand-alone transcutaneous electrotherapy interventions (13–16, 37, 44–46), three studies included only mixed interventions (38, 40, 42) (electrotherapy *plus* an additional intervention), and three studies evaluated both stand-alone and mixed interventions (39, 41, 43) Study characteristics are provided in Table 2, while the results are presented, per outcome, in Tables 3–7.

**Table 2:** Characteristics of included studies.

Study	Participants	Study arms	Treatment	Duration	Outcomes	Time points
Albornoz-Cabello et al. (37)	Individuals aged 20–65 with CLBP (with or without radiating pain), with pain over 40 mm on the VAS, and a score <45 on the Personal Psychological Apprehension Scale (*n* = 64) Age: 51 (11.93) Sex: 31.25% male BMI: 27.87 (4.57)	1. Transregional IFC protocol [IFC] (*n* = 44) 2. Usual care [UC] (*n* = 20)	1. IFC: Electrodes placed at L1 & L5. 4,000 Hz carrier frequency, 65 Hz amplitude modulated frequency, 1:1 swing pattern. Intensity increased to produce “pins and needles” sensation w/out visible muscle twitch. 2. Relaxation massage, soft-tissue techniques, active or passive lumbar spine mobilization	1. 25 min/session, 1 session/day for 2 weeks 2. 25 min/session, 1 session/day for 2 weeks	Pain (VAS); disability (ODI)	Baseline; immediately after last treatment
Alrwaily et al. (38)	Individuals aged 18–60 with CLBP, BMI < 34, NPRS ≥3, MODI ≥20% (*n* = 30; 4 dropouts) Age: STAB = 38.33 (11.3); SNMES = 33.4 (9) Sex: STAB = 4 men, 11 women; SNMES = 7 men, 8 women BMI: STAB = 25.89 (3.8); SNMES = 4.2 (1.9)	1. Stabilization exercises (*n* = 13) 2. Stabilization exercises + NMES [SNMES] (*n* = 13)	1. 5–6 cycles of cat-camel, followed by supine & standing postures with abdominal bracing, bridging, supine leg-lifts, and side-support exercises. Positions were held for 4–8 s and progressed once target reps were reached. 2. NMES: EMPI 300 unit; 75 pps; 250 us; 4 s ramp time, 6 s stimulation period, 50 s rest. Electrodes applied to lumbar paraspinal muscles bilaterally. Participants were told that the stronger the current, the better. They performed active trunk extension when they felt the current ramp up, and rested when the current ramped down. This was followed by the stabilization program described above.	1. 20 min/session, 2×/week for 6 weeks 2. 40 min/session, 2×/week for 6 weeks	Pain (triple NPRS); disability (MODI); fear-avoidance for physical activity and work (FABQ-PA & FABQ-W); paraspinal muscle strength (Biodex 3 Pro dynamometer); self-reported NMES tolerability (NMES group only)	Baseline; during final session
Batistella et al. (15)	Sedentary women with CNSLBP (*n* = 24; 1 exclusion) Age: 21.5 (2.3) BMI: 22.3 (3.8)	1. Russian current [RC] (*n* = 11) 2. Control [C] (*n* = 12)	1. Russian current: two-channel; Ibramed unit; 2,500 Hz frequency with 50 Hz modulation; 2 s ramp, 8 s on, 12 s off time. Electrodes were placed 3 cm lateral to the SPs of T12 & S1. Intensity was increased so that a non-painful muscular contraction was perceived through palpation. 2. No intervention. Group was asked to not perform any physical or medicinal activities during the study period.	1. 20 min/session, 3 sessions/week for 4 weeks. 2. Two months	Pain (VAS); pain pressure threshold (Kratos algometer); disability (ODI); paraspinal muscle endurance; resting multifidus thickness (US)	Baseline; post-intervention; 1 month post-intervention
Caldas et al. (39)	Individuals aged 18–60 years with CLBP, BMI <28, NPRS ≥3 (*n* = 56; 12 withdrawals) Age: C = 28.36 (9.62); CT = 29 (8.09); BTENS = 29.63 (9.54); COMB = 28.54 (8.69) Sex: 54.5% female	1. Cryotherapy [CT] (*n* = 11) 2. Burst TENS [BTENS] (*n* = 11) 3. Combination = Cryotherapy + burst TENS [COMB] (*n* = 11) 4. Control [C] (*n* = 11)	1. 700 mg ice (Thermomatic TH3 machine) + pain education 2. Burst TENS: KLD Sonophasys, 4 Hz, 240 us, maximum intensity to generate visible muscle contraction without generating pain/discomfort due to stimulation + pain education 3. Combined TENS: KLD Sonophasys, 4 Hz, 240 us, maximum intensity to generate visible muscle contraction without generating pain/discomfort due to stimulation; followed by cryotherapy + pain education 4. Sham TENS: (KLD Sonophasys, 4 Hz, 240 us, minimum therapeutic dose) + pain education	1. 10 min pain education, 30 min cryotherapy, 3×/week for 4 weeks 2. 10 min pain education, 30 min burst TENS, 3×/week for 4 weeks 3. 10 min pain education, 30 min combined burst TENS + cryotherapy (specific breakdown not provided), 3×/week for 4 weeks 4. 10 min pain education, 30 min sham TENS, 3×/week for 4 weeks	Functional capacity (Roland-Morris); quality of life (SF-36); sit-to-stand test; pain (VAS); pain pressure threshold (Wagner Force Ten)	Functional capacity, QoL, sit-to-stand test = baseline, week 4 (post-intervention) Pain & ppt = baseline, immediately after 1st treatment, week 1, week 2, week 3, week 4 (post-intervention)
Depaoli-Lemos et al. (40)	Individuals 18–70 years with CLBP (*n* = 48) Age: EXCITENS = 52.5 (12.42); EXCIEA = 45.37 (13.51); EXCI = 50.81 (12.96) Sex: EXCITENS = 3 male, 13 female; EXCIEA = 6 male, 10 female; EXCI = 5 male, 11 female BMI: EXCITENS = 28.95 (4.32); EXCIEA = 27.36 (4); EXCI = 27.29 (3.11)	1. Exercise [EXCI] (*n* = 16) 2. Exercise + TENS [EXCITENS] (*n* = 16) 3. Exercise + electroacupuncture [EXCIEA] (*n* = 16)	1. Exercise: stretches for the posterior chain muscles, isometric strengthening exercises for the core (supine bridge, single leg supine bridge, side plank, prone plank). 2. Exercise: as above. Followed by TENS: two-channel Ibramed unit, 250 us; 10 Hz. Four electrodes placed bilaterally on the paravertebral musculature; current intensity increased to participant's tolerance. 3. Exercise: as above. Followed by electroacupuncture: Sikuro DS100jr unit, two channels with four stimulator cables; 25 × 40 mm needles used at B22 (L1) & B26 (L5). Signal 1, continuous pulse train, 10 Hz. Intensity was increased to tolerance.	1. 50 min/session, 3 sessions/week for 4 weeks 2. 50 min/session, 3 sessions/week for 4 weeks (including 20 min TENS) 3. 50 min/session, 3 sessions/week for 4 weeks (including 20 min EA)	Pain (VAS); function (Roland-Morris); posterior chain flexibility (Wells Bench test); static trunk flexion endurance; static trunk extension endurance (Sorenson test); side bridge	Baseline, post-intervention, 1-month post-intervention
Dimer daLuz et al. (41)	Females 18–36 years with CNSLBP, not engaging in regular physical activity, VAS > 4/10 (*n* = 32; 2 withdrawals) Age: CORE = 26.4 (3.41); NMES = 25.5 (5.28); COMB = 27.1 (4.95) BMI: CORE = 22.56 (3.35); NMES = 27.74 (5.36); COMB = 25.79 (5.5)	1. CORE exercise group (*n* = 10) 2. NMES group (*n* = 10) 3. Combined (COMB) group (*n* = 10)	1. CORE: 4 exercises per session, each maintained for 10 s. 10 reps were performed with a 20 s rest between sets and 1 min rest between exercises. Exercises: prone bridge, supine, side bridge, bird-dog + progressions. 2. NMES: Program targeted gluteus maximums and medius, rectus abdominus, transversus abdominus. Pre-calibrated mid-frequency 10-channel Neruodyn unit; 2,500 Hz carrier frequency; 1 s ramp time, 10 s on, 20 s off. Negative electrodes were positioned on the motor points, and positive electrodes were positioned proximal or distal to the muscle belly. Stimulus started at 5 Hz for 5 min, increased to 35 Hz for 10 min, and then 80 Hz for 10 min. Intensity was increased to the max needed to produce a strong, visible muscle contraction without causing discomfort to the participant. 3. COMB. ES was synchronized to CORE exercises.	1. ∼ 7 min of training time (not including rest periods)/session, 3×/week for 4 weeks 2. 25 min/session 3×/week for 4 weeks 3. 25 min/session 3×/week for 4 weeks	Pain (VAS); disability (ODI); function (Roland-Morris); hamstring flexibility; static trunk endurance; back extensor endurance (Sorenson test); side bridge; prone instability	Baseline, post-intervention, 6-months post-intervention
Elserty et al. (42)	CLBP patients 20–50 years, without radiating pain (*n* = 120; 75 withdrawals) Age: EXCI = 34.93 (8.56); FTENS = 35.73 (8.01); ATENS = 35.13 (8.4) Sex: 14 men (31%), 31 women (69%)	1. Exercise only [EXCI] (*n* = 15) 2. Fixed TENS + exercise [FTENS] (*n* = 15) 3. Adjusted TENS + exercise [ATENS] (*n* = 15)	1. EXCI: progression of bridging and quadruped exercises 2. FTENS: symmetric biphasic current, pulse duration 100 ms, frequency 120 Hz. Electrodes were placed bilaterally at the level of the lumbar vertebrae. Amplitude was increased until patients felt a comfortable tingling sensation + EXCI 3. ATENS: symmetric biphasic current, pulse duration 100 ms, frequency 120 Hz. Electrodes were placed bilaterally at the level of the lumbar vertebrae. Amplitude was increased until patients felt a comfortable tingling sensation. At 5-min intervals, participants were asked if the sensation had faded; if so, the amplitude was increased until the tingling reappeared + EXCI	1. Length of sessions not described, 3×/week for 4 weeks 2. TENS for 40 min + EXCI, 3×/week for 4 weeks 3. TENS for 40 min + EXCI, 3×/week for 4 weeks	Pain (VAS); disability (ODI); spinal ROM in flexion/extension (dual inclinometer)	Baseline, post-intervention
Kofotolis et al. (43)	Women with CLBP, who had both unsuccessful resting periods for 6 months prior and unsuccessful previous therapy (*n* = 92; 4 withdrawals) Age: RS = 41 (5.5); TENS = 41.2 (5); COMB = 37.5 (8.6); PTENS = 42.2 (7.8) BMI: RS = 24.9 (1.2); TENS = 24.6 (1); COMB = 24.3 (1.4); PTENS = 23.8 (1.7)	1. Rhythmic stabilization [RS] (*n* = 23) 2. TENS (*n* = 23) 3. Rhythmic stabilization + TENS [COMB] (*n* = 21) 4. Placebo TENS [PTENS] (*n* = 21)	1. RS: Alternating isometric trunk flexion-extension exercises against resistance for 10 s. Participants performed 3 × 15 at max resistance provided by a physical therapist. 30 s rest between reps (each pattern), 60 s rest between sets. Intensity progression was made according to PNF principles based on participants’ mobility progress. 2. TENS: 120 Z unit (ITO, Tokyo, Japan); pulse duration 200 us, frequency 4 Hz, intensity “strong but comfortable”. Four rubber electrodes (2 × 3 cm) from a dual channel unit were applied to the thoracolumbar fascia and 10 cm proximal, along the midline of the muscle. 3. TENS followed by RS. 4. The same unit was used as for TENS, but the internal circuit was disconnected by the manufacturer.	1. 30–45 min, 5×/week for 4 weeks 2. 40–45 min, 5×/week for 4 weeks 3. 20 min TENS, 5 min rest, 20 min RS (total 45 min), 5×/week for 4 weeks 4. 40–45 min, 5×/week for 4 weeks	Pain (Borg Verbal Rating Pain Scale); disability (ODI); trunk ROM in flexion/extension (flexicurve technique); dynamic flexion endurance (curl-up); dynamic extension endurance (modified Sorenson back extension test); Static flexion endurance (curl-up); static extension endurance (modified Sorenson back extension test)	Baseline, post-intervention, 1-month post, 2-month post
LaraPalomo et al. (16)	Individuals 18–65 years with CLBP, ≥4 on Roland-Morris, not undergoing another physical therapy intervention, with an inability to achieve lumbar muscle flexion-relaxation in trunk flexion (*n* = 62; 1 lost to follow-up) Age: 48 (15) Sex: 67.8% female	1. IFC electro-massage [IFC] (*n* = 30) 2. Superficial massage [SM] (*n* = 31)	1. 4,000 Hz carrier frequency, 80 Hz amplitude modulation, constant voltage. Bipolar application with 2 electrodes to which sponges were fitted. The sponges moved over the lumbar and thoraco-lumbar regions. Intensity was increased to between 30 and 50 mA, always below pain threshold. 2. Massage consisting of effleurage, petrissage, and skin rolling.	1. 30 min/session, 2×/week for 10 weeks 2. 20 min/session, 2×/week for 10 weeks	Pain (VAS); disability (ODI); function (Roland-Morris); kinesiophobia (TSK); quality of life (SF-36); isometric abdominal resistance (McQuade test); side bridge test; trunk anteflexion ROM	Baseline, post-intervention.
Pelegrini et al. (14)	Patients with low back pain for at least 3 months, aged 19–70 years, (*n* = 24, no dropouts) Age: 20.4 (1.8) y Sex: 100% women	1. Aussie current group [AC] (*n* = 12) 2. Control group [C] (*n* = 12)	1. Treatment with Neurodyn Ibramed device. Frequency 1 kHz, modulation 50 Hz, burst duration 4 ms, ramp time 1 s, maintained for 8 s, rest 10 s. Intensity increased until visible (but not painful) involuntary muscular contraction was achieved. If accommodation phenomenon was achieved, the therapist increased the amplitude of the current 2. Participants agreed to not participate in any therapeutic activity for 4 weeks.	1. 20 min, 3×/week for 4 weeks 2. No treatment for 4 weeks	Pain (VAS, McGill Pain Questionnaire); disability (ODI); trunk extensor endurance (horizontal board test aka Sorenson test); resting multifidus thickness (ultrasound)	Baseline, post-intervention, 1-month post-intervention
Topuz et al. (44)	Individuals 18–70 years with CLBP (*n* = 60; 5 dropouts) Age: 44.11 (12.21) Sex: 19 men, 41 women	1. Placebo TENS [PTENS] (*n* = 12) 2. Conventional TENS [CTENS] (*n* = 15) 3. Low-frequency TENS [LFTENS] (*n* = 15) 4. Percutaneous neuromodulation therapy [PNT] (*n* = 13)	1. Trio 300 TENS unit was used. Four medium-sized (2 cm× 2 cm) carbon impregnated rubber cutaneous electrodes were placed bilaterally in a standard dermatomal pattern over the most painful lumbar region. No electrical stimulation was applied. 2. Trio 300 TENS unit was used—symmetric, biphasic rectangular pulses with 100 us duration, 80 Hz frequency. Four medium-sized (2 cm× 2 cm) carbon impregnated rubber cutaneous electrodes were placed bilaterally in a standard dermatomal pattern over the most painful lumbar region. Current intensity was increased up to patients’ perception of paraesthesia. 3. Trio 300 TENS unit was used—symmetric, biphasic rectangular pulses with 100 us duration, 4 Hz frequency. Four medium-sized (2 cm × 2 cm) carbon impregnated rubber cutaneous electrodes were placed bilaterally in a standard dermatomal pattern over the most painful lumbar region. Current intensity was increased to maximum tolerated amplitude without muscle contractions. *All subjects were told that they might or might not perceive the stimulation, that stimulation was sometimes below a persons’ threshold of perception, and this should not be of concern. 4. IC 4,017 unit (ITO Corp., Japan) that generates unipolar square-wave pulses with 100 us duration. Subjects received low-frequency stimulation (4 Hz); current intensity was increased to maximum tolerated amplitude to produce the highest electrical “tapping” sensation without muscular contractions. Four 32 gauge stainless steel acupuncture-like needle electrodes were placed symmetrically into the soft tissue to a depth of 2–4 cm in a standard dermatomal pattern over the most painful lumbar region.	1. 20 min/session, 5×/week for 2 weeks. 2. 20 min/session, 5×/week for 2 weeks. 3. 20 min/session, 5×/week for 2 weeks. 4. 20 min/session, 5×/week for 2 weeks.	Current pain (VAS); activity pain (VAS); functional status (LBPOS); disability (ODI); quality of life (SF-36); depression (BDI)	Baseline, post-intervention
Weissenfels et al. (13)	Patients with nonspecific chronic low back pain for at least 3 months (aged 40–70 years) Age: WB-EMS = 54.6 (5.7); CG = 59.4 (7.7) Total body fat %: WB-EMS = 25.4 (9.3) for men, 31.3 (8.4) for women. C: 29.9 (5.4) for men, 35.5 (9.1) for women	1. WB-EMS (*n* = 15) 2. C (*n* = 15)	1. Bipolar electric current with a frequency of 85 Hz, an impulse width of 350 µs, a rectangular mode and an interval of 6 s stimulation and 4 s of rest once a week for 20 min. During the stimulation phase, participants performed low-amplitude movements specifically dedicated to LBP. The participants completed one to three sets with six repetitions of six easy movements in a minor range of motion (e.g., dynamic squatting with knee ankle ≥120°) to keep the effect of the voluntary exercise itself as low as possible. The intensity of the stimulation was regulated using the BORG CR 10 scale. Subjects were requested to exercise at a rate of perceived exertion (RPE) between “hard (5)” and “very hard (7)”. In the first session current intensity was individually adapted in close interaction with the participants and saved to generate a fast and valid setting during the following sessions. 2. The CG was asked to maintain its usual lifestyle. Participants of this group were regularly contacted by phone and asked about their current status and lifestyle changes.	1. 12 min for 1st session, 14 for 2nd, 16 for 3rd, 18 for 4th, 20 min for last 8 weeks. 1 session/week for a total of 12 weeks 2. No specific intervention for 12 weeks	Average pain Intensity (NPRS); isometric trunk flexion strength; isometric trunk extension strength	For pain: baseline (4 weeks prior to intervention); post-intervention (last 4 weeks of intervention) For strength: baseline, post-intervention
Weissenfels et al. (45)	Patients with nonspecific chronic low back pain for at least 3 months (aged 40–70 years) Age: WB-EMS = 57.4 (7.6); CT = 54.4 (7.4) Sex: WB-EMS = 17 m, 28w; CT = 20 m, 35w Total body fat %: WB-EMS = 23.4 (4.3) for men, 35 (8.2) for women; CT = 25.1 (8.9) for men, 32.9 (8.7) for women	1. WB-EMS (*n* = 55) 2. CT (*n* = 55)	1. Bipolar electric current with a frequency of 85 Hz, an impulse width of 350 µs, a rectangular mode and an interval of 6 s stimulation and 4 s of rest once a week for 20 min. Participants performed the following exercises: squat with latissimus pulleys, butterfly reverse, straight pullovers with trunk flexion, standing trunk flexion; one-legged stand with biceps curl; side step with weight shift and biceps curl. With the exception of the first week, participants were instructed to perform exercises at an RPE between “strong” and “very strong” 2. Participants performed conventional back strengthening/core stabilization exercises described in various meta-analyses. After 15 min warm-up, 10 trunk strengthening exercises were performed in a circle for 30 min. The circle repeated twice, with 50s work and 25 s break between exercises.	1. 12 min for 1st session, 14 for 2nd, 16 for 3rd, 18 for 4th, 20 min for last 8 weeks. 1 session/week for a total of 12 weeks 2. 45 min per week for 12 weeks	Average pain Intensity (NPRS); isometric trunk flexion strength (Back-Check 607); isometric trunk extension strength (Back-Check 607)	For pain: baseline (4 weeks prior to intervention); post-intervention (last 4 weeks of intervention) For strength: baseline, post-intervention
Yaksi et al. (46)	CLBP patients 18–65 years, including with lumbar disc disease without radicular compression (*n* = 74; 1 lost to follow-up) Age: 43.3 (11.3) Sex: 64.4% female BMI: 28.6 (4.6)	1. Conventional TENS [CTENS] (*n* = 25) 2. Burst TENS [BTENS] (*n* = 25) 3. Placebo TENS [PTENS] (*n* = 23)	1. TENS Intellect Advanced unit (Chattanoga, France). Four electrodes were used. Two electrodes (active) were attached 1.5 cm laterally to the vertebrae at the L2-L4 level, and the other two electrodes (passive) were attached 3 cm distal to the active electrodes. 60–80 Hz; pulse width 50–80 us; intensity 10–30 mA. Intensity was increased to a moderate level (below patient discomfort) 2. TENS Intellect Advanced unit (Chattanoga, France). Four electrodes were used. Two electrodes (active) were attached 1.5 cm laterally to the vertebrae at the L2–L4 level, and the other two electrodes (passive) were attached 3 cm distal to the active electrodes. Baseline low frequency current (1–4 Hz) with high frequency trains (50–100 Hz). Intensity was increased to a moderate level (below patient discomfort). 3. TENS Intellect Advanced unit (Chattanoga, France). Four electrodes were used. Two electrodes (active) were attached 1.5 cm laterally to the vertebrae at the L2-L4 level, and the other two electrodes (passive) were attached 3 cm distal to the active electrodes. No current was applied.	1. 30 min/session, 5 days/week for 3 weeks 2. 30 min/session, 5 days/week for 3 weeks 3. 30 min/session, 5 days/week for 3 weeks	Daytime & nighttime pain (VAS); neuropathic pain (DN4); disability (mODI); depression (BDI); peripheral neuropathy, nerve & nerve root lesions (SSR)	Baseline, post-intervention, 3 months post-intervention (VAS); Baseline, 3 months post-intervention (all other outcomes)

*Supplementary information about study methods.

**Table 3:** Outcome: pain.

Study	Groups	Outcome/tool	Result: post-intervention	Result: 1-month post	Result: ≥2-month post
Albornoz-Cabello et al. (37)	1. Transregional IFC protocol [IFCG] (*n* = 44) 2. Usual care (*n* = 20)	Pain (VAS)	IFC group reduced pain by 49.90 (16.85), ***p*** < **0.001**. Usual care group reduced pain by 37.75 (19.63), ***p*** < **0.001**. IFC group had greater pain reduction than usual care group (MD = 11.34, 95% CI = 1.77, 20.91, ***p* = 0.032**).	NA	NA
Alrwaily et al. (38)	1. Stabilization exercises (*n* = 13) 2. Stabilization exercises + NMES [SNMES] (*n* = 13)	Pain (triple NPRS)	STAB group improved from 4.44 (1.8) to 2.07 (1.1), Δ-2.37, ***p*** < **0.05**. SNMES group improved from 4.20 (1.9) to 2.34 (1.5), Δ-1.86, ***p*** **<** **0.05**. Δ in pain was not significantly greater in the STAB group (MD = −0.27, 95% CI = −1.24, 0.70).	NA	NA
Batistella et al. (15)	1. Russian current [RC] (*n* = 11) 2. Control [C] (*n* = 12)	Pain (VAS)	RC group improved from 6 (4.5/7.3) to 4 (2/3), ***p* = 0.0069**. C group improved from 6 (5.9/7) to 4 (4/5.2), ***p* = 0.0248**. There was a significant between-group difference (***p* = 0.0483**).	RC group: 3.5 (2/5). C group: 5 (4/6). There was no significant between-group difference.	NA
Caldas et al. (39)	1. Cryotherapy [CT] (*n* = 11) 2. Burst TENS [BTENS] (*n* = 11) 3. Combination = Cryotherapy + burst TENS [COMB] (*n* = 11) 4. Control [C] (*n* = 11)	Pain (VAS)	CT group improved from 4.23 (1.2) to 1.21 (1.3), ***p*** < **0.**05. BTENS group improved from 4.74 (0.83) to 0.68 (0.44), ***p*** < **0.05**. COMB group improved from 4.13 (1.62) to 1.2 (1.25), ***p*** < **0.**05. C group improved from 4.65 (1.31) to 0.95 (0.95), ***p*** < **0.05**. There were no significant between-group differences.	NA	NA
Depaoli-Lemos et al. (40)	1. Exercise + TENS [EXCITENS] (*n* = 16) 2. Exercise + electroacupuncture [EXCIEA] (*n* = 16) 3. Exercise [EXCI] (*n* = 16)	Pain (VAS)	EXCITENS group improved from 8.88 (1.03) to 3.56 (2.85), ***p*** < **0.01**. EXCIEA group improved from 8.75 (0.93) to 1.25 (1.18), ***p*** < **0.01**. EXCI group improved from 9.19 (0.83) to 3.31 (2.85), ***p*** < **0.01**. There were significant between-group differences (***p*** = **0.02**) at post-intervention between EXCITENS & EXCIEA, and between EXCIEA & EXCI (both in favour of EXCIEA)	EXCITENS: 4.0 (3.18). Significant improvement from baseline to 1-month post (***p*** < **0.01**). EXCIEA: 0.94 (1.06). Significant improvement from baseline to 1-month post (***p*** < **0.01**). EXCI: 3.81 (2.29). Significant improvement from baseline to 1-month post (***p*** < **0.01**). There were significant between- group differences (***p*** = **0.001**) at 1-month post between EXCITENS & EXCIEA, and between EXCIEA & EXCI (both in favour of EXCIEA).	NA
Dimer daLuz et al. (41)	1. CORE exercise group (*n* = 10) 2. NMES group (*n* = 10) 3. Combined (COMB) group (*n* = 10)	Pain (VAS)	CORE group improved from 6.4 (0.84) to 2.1 (2.28), ***p*** < **0.05**. NMES group improved from 6.8 (0.42) to 2.9 (1.28), ***p*** < **0.05**. COMB group improved from 6.6 (1.07) to 0.4 (0.96), ***p*** < **0.05**. There was a significant difference between NMES & COMB (***p*** < **0.05**), in favor of COMB.	NA	At 6-months post: CORE: 4.2 (2.15). NMES: 4.2 (1.47). COMB: 2.6 (2.31). There were no significant between-group differences.
Elserty et al. (42)	1. Exercise only [EXCI] (*n* = 15) 2. Fixed TENS + exercise [FTENS] (*n* = 15) 3. Adjusted TENS + exercise [ATENS] (*n* = 15)	Pain (VAS)	EXCI group changed from 7.33 {1.05} to 3.77 {0.77}. FTENS group changed from 7.6 {1.02} to 2.5 {1.03}. ATENS group changed from 7.5 {0.95} to 2.17 {0.92}. There was a significant between-group difference at post- intervention (***p*** < **0.0001**). Fisher least-significant difference test revealed greater improvements in both TENS groups compared to the EXCI group. *{} denotes Standard Error*	NA	NA
Kofotolis et al. (43)	1. Rhythmic stabilization [RS] (*n* = 23) 2. TENS (*n* = 23) 3. Rhythmic stabilization + TENS [COMB] (*n* = 21) 4. Placebo TENS [PTENS] (*n* = 21)	Pain (Borg Verbal Rating Pain Scale)	RS group improved from 2.1 (0.8) to 1.6 (0.4), (***p*** < **0.05**). TENS group changed from 2.3 (0.4) to 2.2 (0.4), *p* > 0.05. COMB group changed from 1.9 (0.6) to 1.7 (0.5), *p* > 0.05. PTENS group changed from 2.1 (0.7) to 2 (0.4), *p* > 0.05. There were significant differences (***p*** < **0.05**) between RS & PG, RS & TENS (both in favor of RS), TENS & COMB (in favor of COMB).	RS: 1.4 (0.5). There was a significant (***p*** < **0.05**) improvement from baseline to 1-month post-intervention. TENS: 2.1 (0.6). COMB: 1.6 (0.4). PTENS: 2 (0.4). There were significant differences (***p*** < **0.05**) between RS & PG, RS & TENS (both in favor of RS), TENS & COMB (in favor of COMB).	At 2-months post: RS: 1.2 (0.3). There was a significant (***p*** < **0.05**) improvement from baseline to 2-months post-intervention. TENS: 2 (0.6). COMB: 1.4 (0.4). PTENS: 1.9 (0.4). There were significant differences (***p*** < **0.05**) between RS & PG, RS & TENS (both in favor of RS), TENS & COMB, COMB & PG (both in favor of COMB).
LaraPalomo et al. (16)	1. IFC electro-massage [IFC] (*n* = 30) 2. Superficial massage [SM] (*n* = 31)	Pain (VAS)	IFC group had a pain decrease of 1.67 (95% CI = 0.94, 2.39), ***p*** = **0.001**. SM group had a pain decrease of 0.45 (95% CI = −0.06, 0.96), *p* > 0.05. IFC group had greater pain reduction than SM group (MD = −1.06, 95% CI = −1.91, −0.22, ***p*** = **0.001**).	NA	NA
Pelegrini et al. (14)	1. Aussie current group [AC] (*n* = 12) 2. Control group [C} (*n* = 12)	Pain (VAS)	AC group improved from 67.7 (11.1) to 41.5 (15.99), ***p*** = **0.0012,** ES = −1.9. C group changed from 52.92 (13.56) to 43.75 (14.0), *p* > 0.05, *ES not reportable due to error in manuscript*. There was a significant between-group difference at baseline (***p*** = **0.0063**, ES = 1.16). The ACG had significantly higher pain at baseline. There were no significant between-group differences at post-intervention (*p* = 0.3643).	AC group: 32.6 (26.65). There was a significant improvement from baseline to 1-month post-intervention (***p*** = 0.0012, ES = −1.72). C group: 55.0 (9.05). There was a significant between-group difference at 1-month post-intervention (***p*** = **0.0147,** ES = −1.18) in favour of AC group.	NA
Pelegrini et al. (14)	1. Aussie current group [ACG] (*n* = 12) 2. Control group [C} (*n* = 12)	Pain (McGill Pain Questionnaire)	AC group improved from 36.5 (34/43.5) to 31.5 (29.0/35.0), ***p*** = **0.0486.** C group changed from 37.0 (31.7/41.2) to 35.0 (32.7/42.0), *p* = 0.8290. There was no significant between-group difference (0.1780).	AC group: 35.0 (33.0/46.2). C group: 34.5 (32.7/40.0). There was no significant between-group difference (0.2764).	NA
Topuz et al. (44)	1. Placebo TENS [PTENS] (*n* = 12) 2. Conventional TENS [CTENS] (*n* = 15) 3. Low-frequency TENS [LFTENS] (*n* = 15) 4. Percutaneous neuromodulation therapy [PNT] (*n* = 13)	Current pain (VAS); activity pain (VAS)	Current Pain PTENS group increased by 0.16 (1.11), *p* > 0.05. CTENS group decreased by −2.8 (2.0), ***p*** < **0.05**. LFTENS group decreased by −2.6 (1.4), ***p*** < **0.05**. PNT group decreased by −3.61 (1.98), ***p*** < **0.05**. There were significant between-group differences in score changes (***p*** < **0.05**) for CTENS vs. PTENS, LFTENS vs. PTENS, PNT vs. PTENS (all in favor of the active intervention). Activity Pain PTENS group decreased by −0.16 (0.83), *p* > 0.05. CTENS group deceased by. −2.5 (1.45), ***p*** < **0.05**. LFTENS group decreased by −2.15 (1.18), ***p*** < **0.05**. PNT group decreased by −4.07 (1.75), ***p*** < **0.05.** There were significant between-group differences in score changes (***p*** < **0.05**) for CTENS vs. PTENS, LFTENS vs. PTENS, PNT vs. PTENS (all in favor of the active intervention), CTENS vs. PNT, LFTENS vs. PNT (both in favor of PNT).	NA	NA
Weissenfels et al. (13)	1. WB-EMS (*n* = 15) 2. C (*n* = 15)	Average pain Intensity (NPRS)	WB-EMS group improved −0.74 (0.87) from baseline to post-intervention **(*p*** < **0.01**). C group improved −0.08 (0.88) from baseline to post-intervention. There was a significantly greater improvement in the WB-EMS group (***p*** = **0.028,** ES = 0.754).	NA	NA
Weissenfels et al. (45)	1. WB-EMS (*n* = 55) 2. CT (*n* = 55)	Average pain Intensity (NPRS)	WB-EMS group improved −0.60 (0.96) from baseline (***p*** < **0.001**). CT group improved 0.85 (0.97) from baseline (***p*** **<** **0.001**). There was no significant between-group difference in score changes over time.	NA	NA
Yaksi et al. (46)	1. Conventional TENS [CTENS] (*n* = 25) 2. Burst TENS [BTENS] (*n* = 25) 3. Placebo TENS [PTENS] (*n* = 23)	Daytime pain (VAS); night-time pain (VAS); neuropathic pain (DN4)	Daytime Pain CTENS group improved by −2.2 (1.9), ***p*** < **0.001**. BTENS group improved by −3.9 (2.4), ***p*** < **0.001**. PTENS group improved by −2.0 (1.7), ***p*** < **0.001**. There was a significant between-group difference in score changes in (***p*** = **0.012**). Mann–Whitney *U*-test with Bonferonni correction revealed a significant difference between BTENS & PTENS (***p*** = **0.007**) in favor of BTENS. Night-time Pain CTENS group improved by −1.6 (1.9), ***p*** = **0.003**. BTENS group improved by −2.8 (2.9), ***p*** < **0.001**. PTENS group improved by −2.3 (2.4), ***p*** < **0.001**. There were no significant between group differences. *neuropathic pain not evaluated	NA	At 3-months post: Daytime Pain CTENS group improved by −2.5 (2.7), ***p*** < **0.001** from baseline. BTENS group improved by −1.3 (2.7), ***p*** < **0.001** from baseline. PTENS group improved by −1.6 (2.0), ***p*** = **0.016** from baseline. There were no significant between group differences. Night-time Pain CTENS group improved by −1.5 (2.4) ***p*** = **0.003** from baseline. BTENS group improved by −2.4 (2.7), ***p*** < **0.001** from baseline. PTENS group improved by −1.8 (2.4), ***p*** < **0.001** from baseline. There were no significant between-group differences. Neuropathic Pain CTENS group improved from 5.1 (1.3) to 3.4 (2.2), ***p*** < **0.001**. BTENS group improved from 5.4 (1.5) TO 2.5 (2.3), ***p*** < **0.001**. PTENS group improved from 5.0 (1.1) to 3.1 (2.1), ***p*** < **0.001**. There were no significant between-group differences.

The bold values represent significant findings.

**Table 4:** Outcome: disability.

Study	Groups	Outcome/tool	Result: post-intervention	Result: 1-month post	Result: ≥2-month post
Albornoz-Cabello et al. (37)	1. Transregional IFC protocol [IFCG] (*n* = 44) 2. Usual care (*n* = 20)	ODI	IFC group reduced disability by 34.68 (16.24), ***p*** < **0.001**. UC group reduced disability by 21.30 (14.01), ***p*** < **0.001**. IFC group had greater r10eduction in disability than usual care group (MD = 13.38, 95% CI = 4.97, 21.78, ***p* = 0.002**)	NA	NA
Alrwaily et al. (38)	1. Stabilization exercises (*n* = 13) 2. Stabilization exercises + NMES [SNMES] (*n* = 13)	mODI	STAB group improved from 30.80 (10.2) to 12.81 (5.2), Δ-17.99, ***p*** < **0.05**. SNMES group improved from 30.52 (7.8) to 14.49 (10.2), Δ-16.03, ***p*** < **0.05**. Δ in disability was not significantly greater in the STAB group (MD = −1.68, 95% CI = −7.88, 4.19).	NA	NA
Batistella et al. (15)	1. Russian current [RCG] (*n* = 11) 2. Control [CG] (*n* = 12)	ODI	RC group improved from 12 (10/12) to 8 (5/14). *Statistical significance not reported*. C group changed from 15 (12.9/18) to 12 (11.5/16), *p* > 0.05. There was no significant between-group difference.	RC group: 12 (8/22). C group: 16 (12/17). There was no significant between-group difference.	NA
Caldas et al. (39)	1. Cryotherapy [CT] (*n* = 11) 2. Burst TENS [BTENS] (*n* = 11) 3. Combination = Cryotherapy + burst TENS [COMB] (*n* = 11) 4. Control [C] (*n* = 11)	RMDQ	CT group improved from 8.63 (3.52) to 4.0 (3.46), ***p*** < **0.05**. BTENS group improved from 10.0 (3.87) to 4.27 (2.72), ***p*** < **0.05**. COMB group improved from 8.27 (2.53) to 4.09 (2.25), ***p*** < **0.05**. C group improved from 8.72 (4.33) to 4.09 (3.78), ***p*** < **0.05**. There were no significant between-group differences.	NA	NA
Depaoli-Lemos et al. (40)	1. Exercise + TENS [EXCITENS] (*n* = 16) 2. Exercise + electroacupuncture [EXCIEA] (*n* = 16) 3. Exercise [EXCI] (*n* = 16)	RMDQ	EXCITENS group improved from 16.63 (3.96) to 10.31 (7.13), ***p* = 0.001.** EXCIEA group improved from 16.5 (4.41) to 4.44 (5.67), ***p* = 0.001**. EXCI group improved from 19.81 (3.23) to 9.88 (5.62), ***p* = 0.001**. There were significant between-group differences at post-intervention (***p* = 0.02**) between EXCITENS & EXCIEA, and EXCITENS & EXCI.	EXCITENS: 9.56 (7.57). Significance improvement from baseline to 1-month post (***p* = 0.001**). EXCIEA: 3.56 (5.32). Significance improvement from baseline to 1-month post (***p* = 0.001**). EXCI: 10.38 (5.19). Significance improvement from baseline to 1-month post (***p* = 0.001**). There were significant between-group differences (***p* = 0.005**) between EXCITENS & EXCIEA (in favour of EXCIEA), and between EXCITENS & EXCI (in favour of EXCITENS).	NA
Dimer daLuz et al. (41)	1. CORE exercise group [CORE] (*n* = 10) 2. NMES group [NMES] (*n* = 10) 3. Combined (CORE + NMES) group [COMB] (*n* = 10)	ODI, RMDQ	ODI CORE group improved from 15.9 (5.06) to 5.7 (3.19), ***p*** < **0.05**. NMES group improved from 13.4 (4.0) to 6.1 (4.14), ***p*** < **0.05**. COMB group improved from 11.8 (1.93) to 1.2 (0.91), ***p*** < **0.05**. There were significant differences between CORE & COMB (***p*** < **0.05**) in favor of COMB, and between NMES & COMB (***p*** < **0.05**) in favor of COMB. RMDQ CORE group improved from 11.5 (2.71) to 2.6 (3.06), ***p*** < **0.05**. NMES group improved from 10.0 (4.41) to 3.6 (3.02), ***p*** < **0.05**. COMB group improved from 11.9 (2.33) to 0.5 (0.85), ***p*** < **0.05**. There was a significant difference between NMES & COMB (***p*** < **0.05**) in favor of COMB.	ODI: NA RMDQ: NA	ODI At 6-months post: CORE: 8.3 (5.57). Significant improvement from baseline to 6 m post (***p*** < **0.05**). NMES: 8.3 (5.29). COMB: 4.2 (4.05). Significant improvement from baseline to 6 m post (***p*** < **0.05**). There were no significant between-group differences. RMDQ CORE: 4.5 (3.62). Significant improvement from baseline to 6 m post (***p*** < **0.05**). NMES: 5.9 (4.72). Significant improvement from baseline to 6 m post (***p*** < **0.05**). COMB: 1.9 (2.37). Significant improvement from baseline to 6 m post (***p*** < **0.05**). There was a significant difference between NMES & COMB (***p*** < **0.05**) in favor of COMB.
Elserty et al. (42)	1. Exercise only [EXCI] (*n* = 15) 2. Fixed TENS + exercise [FTENS] (*n* = 15) 3. Adjusted TENS + exercise [ATENS] (*n* = 15)	4. ODI	EXCI group changed from 57.57 {12.72} to 22.07 {8.21}. FTENS group changed from 58.94 {12.02} to 18.37 {7.39}. ATENS group changed from 59.85 {11.92} to 15.4 {7.38}. There were no significant between-group differences at post-intervention (*p* = 0.069). *{} denotes Standard Error*	NA	NA
Kofotolis et al. (43)	1. Rhythmic stabilization [RS] (*n* = 23) 2. TENS (*n* = 23) 3. Rhythmic stabilization + TENS [COMB] (*n* = 21) 4. Placebo TENS [PTENS] (*n* = 21)	ODI	RS group improved from 17.1 (2.5) to 12.6 (3.1), ***p*** < **0.05**. TENS group changed from 18.3 (2.3) to 17.4 (2.2), *p* > 0.05. COMB group changed from 17.4 (2.2) to 15.3 (3.1), *p* > 0.05. PTENS group changed from 15.7 (4.7) to 16.7 (2.8), *p* > 0.05. There were significant differences (***p*** < **0.05**) between RS & PTENS, RS & COMB, RS & TENS (all in favour of RS).	RS: 10.0 (1.0). Significant improvement from baseline to 1 m post (***p*** < **0.05**). TENS: 17.1 (1.7). COMB: 14.2 (2.3). Significant improvement in COMB group from baseline to 1m-post (***p*** < **0.05**). PTENS: 16.5 (2.0) There were significant differences (***p*** < **0.05**) between RS & PTENS, RS & COMB, RS & TENS (all in favor of RS), COMB & PTENS, COMB & TENS (both in favor of COMB).	At 2-month post: RS: 9.9 (0.8). Significant improvement from baseline to 2 m post (***p*** < **0.05**). TENS: 16.3 (3.7). COMB: 13.7 (1.9). Significant improvement from baseline to 2m-post (***p*** < **0.05**). PTENS: 15.8 (1.9). There were significant differences (***p*** < **0.05**) between RS & PTENS, RS & COMB, RS & TENS (all in favor of RS), COMB & PTENS, COMB & TENS (both in favor of COMB).
LaraPalomo et al. (16)	1. IFC electro-massage [IFC] (*n* = 30) 2. Superficial massage [SM] (*n* = 31)	ODI; RMDQ	ODI IFC group improved from baseline (MD = 5.47, 95% CI = −0.68, 11.61, *p* > 0.05). SM group improved from baseline (MD = 2.06, 95% CI = −0.36, 4.49, *p* > 0.05). There was a significant between-group difference in score change (MD = −5.2, 95% CI = −10.82, 0.42, ***p* = 0.019**) in favour of IFC. RMDQ IFC group improved significantly from baseline (MD = 2.37, 95% CI = 0.98, 3.75, ***p* = 0.038**). SM group improved from baseline (MD = 0.16, 95% CI = −0.61, 0.93, *p* > 0.05). There was a significant between-group difference in score change (MD = −3.01, 95% CI = −4.53, −1.47, ***p* = 0.006**) in favour of IFC.	ODI: NA RMDQ: NA	ODI: NA RMDQ: NA
Pelegrini et al. (14)	1. Aussie current group [ACG] (*n* = 12) 2. Control group [CG} (*n* = 12)	ODI	AC group changed from 16.0 (13.2/21.5) to 15.0 (10.5/21.5), *p* = 0.1225. C group changed from 14.0 (12.0/16.0) to 12.0 (12.0/16.0), *p* = 0.9394. There was no significant between-group difference (*p* = 0.4345).	AC group: 16.0 (11.5/19.2). C group 13.0 (11.4/16.0). There was no significant between-group difference (*p* = 0.1176).	NA
Topuz et al. (44)	1. Placebo TENS [PTENS] (*n* = 12) 2. Conventional TENS [CTENS] (*n* = 15) 3. Low-frequency TENS [LFTENS] (*n* = 15) 4. Percutaneous neuromodulation therapy [PNT] (*n* = 13)	ODI; LBPOS	ODI PTENS group worsened by 2.16 (3.29), *p* > 0.05. CTENS group improved by −6.60 (5.7), ***p*** < **0.05**. LFTENS group improved by −7.73 (4.26), ***p*** < **0.05**. PNT group improved by −9.53 (4.85), ***p*** < **0.05**. There were significant differences (***p*** < **0.05**) in score changes for CTENS vs. PTENS, LFTENS vs. PTENS, PNT vs. PTENS (all in favor of the active intervention). LBPOS PTENS group improved by 8.33 (5.86), *p* > 0.05. CTENS group improved by 13.55 (9.63), ***p*** < **0.05**. LFTENS group improved by 12.8 (7.0), ***p*** < **0.05**. PNT group improved by 15.38 (12.95), ***p*** < **0.05**. There were significant differences (***p*** < **0.05**) in score changes for CTENS vs. PTENS, LFTENS vs. PTENS, PNT vs. PTENS (all in favor of the active intervention).	ODI: NA LBPOS: NA	ODI: NA LBPOS: NA
Yaksi et al. (46)	1. Conventional TENS [CTENS] (*n* = 25) 2. Burst TENS [BTENS] (*n* = 25) 3. Placebo TENS [PTENS] (*n* = 23)	mODI	NA	NA	At 3-months post: CTENS group improved from 30.6 (8.2) to 26.0 (10.6), ***p* = 0.003**. BTENS group improved from 32.0 (6.3) to 25.4 (9.4), ***p* = 0.001**. PTENS group improved from 32.6 (6.3) to 28.3 (7.9), ***p* = 0.002**. There were no significant between-group differences.

The bold values represent significant findings.

**Table 5:** Outcome: quality of life.

Study	Groups	Outcome/tool	Result: post-intervention
Caldas et al. (39)	1. Cryotherapy (*n* = 11) 2. Burst TENS (*n* = 11) 3. Combination = Cryotherapy + burst TENS (*n* = 11) 4. Control (*n* = 11)	SF-36	Significant improvement (*p* < 0.05) in the following domains: functional capacity (for CTG, BTENSG, CG); limitations by physical aspects (BTENSG, CG); pain (all groups); general health status (CTG, BTENSG, CG); vitality (CTG, CG); social aspects (CTG); limitations by emotional aspects (CTG); mental health (COMBG)
LaraPalomo et al. (16)	1. IFC electro-massage [IFC] (*n* = 30) 2. Superficial massage [SM] (*n* = 31)	SF-36	Significant improvement in IFC group compared to baseline for physical function (*p* = 0.001), physical role (*p* = 0.001), body pain (*p* = 0.001), general health (*p* = 0.021), vitality (*p* = 0.036), social function (*p* = 0.002), emotional role (*p* = 0.049), and mental health (*p* = 0.011). Significant improvement in SM group compared to baseline for physical function (*p* = 0.044). Significant between-group difference in score changes for physical function: 5.57 (−2.27, 13.41), F=16.792, *p* = 0.001; physical role: 7.02 (1.05, 12.98), F=14.830, *p* = 0.001); body pain: 4.72 (−0.28, 9.71), F=11.247, *p* = 0.001 in favour of IFC.
Topuz et al. (44)	1. Placebo TENS [PTENS] (*n* = 12) 2. Conventional TENS [CTENS] (*n* = 15) 3. Low-frequency TENS [LFTENS] (*n* = 15) 4. Percutaneous neuromodulation therapy [PNT] (*n* = 13)	SF-36	Significant worsening (*p* < 0.05) for PTENS in emotional role limitations only. Significant improvement (*p* < 0.05) for CTENS in all domains *except* emotional role limitations. Significant improvement (*p* < 0.05) for LFTENS & PNT across all domains. Significant (*p* < 0.05) between-group difference in score changes in *physical function* for CTENS vs. PTENS, LFTENS vs. PTENS, PNT vs. PTENS (all in favor of the active intervention). Significant (*p* < 0.05) between-group difference in score changes in *social functioning* for CTENS vs. PTENS, LFTENS vs. PTENS, PNT vs. PTENS (all in favor of the active intervention). Significant (*p* < 0.05) between-group difference in score changes in *physical role limitation* for CTENS vs. PTENS, LFTENS vs. PTENS, PNT vs. PTENS (all in favor of the active intervention). Significant (*p* < 0.05) between-group difference in score changes in *physical function* for CTENS vs. PTENS, LFTENS vs. PTENS, PNT vs. PTENS (all in favor of the active intervention). Significant (*p* < 0.05) between-group difference in score changes in *emotional role limitations* for CTENS vs. PTENS, LFTENS vs. PTENS, PNT vs. PTENS (all in favor of the active intervention), CTENS vs. PNT (in favor of PNT). Significant (*p* < 0.05) between-group difference in score changes in *general mental health* for CTENS vs. PTENS, LFTENS vs. PTENS, PNT vs. PTENS (all in favor of the active intervention). Significant (*p* < 0.05) between-group difference in score changes in *vitality* for CTENS vs. PNT, PTENS vs. PNT (both in favor of PNT). Significant (*p* < 0.05) between-group difference in score changes in *bodily pain* for CTENS vs. PTENS, LFTENS vs. PTENS, PNT vs. PTENS (all in favor of the active intervention). Significant (*p* < 0.05) between-group difference in score changes in *general health perception* for CTENS vs. PTENS, LFTENS vs. PTENS, PNT vs. PTENS (all in favor of the active intervention), CTENS vs. PNT, LFTENS vs. PNT (both in favor of PNT).

**Table 6:** Outcome: fear-avoidance beliefs.

Study	Groups	Outcome/tool	Result: post-intervention
Alrwaily et al. (38)	1. Stabilization exercises (*n* = 13) 2. Stabilization exercises + NMES [SNMES] (*n* = 13)	FABQ-W; FABQ-PA	FABQ-W STAB group changed from 12.20 (10.9) to 8.87 (9.6), Δ3.33, *p* > 0.05. SNMES group changed from 11.67 (10.5) to 7.75 (7.6), Δ3.92, *p* > 0.05. There were no significant between-group differences. FABQ-PA STAB group improved from 12.33 (5.5) to 8.41 (6.0), Δ3.92, ***p*** < **0.05**. SNMES group improved from 14.27 (6.5) to 10.75 (4.7), Δ3.52, ***p*** < **0.05**. There were no significant between-group differences.

The bold values represent significant findings.

**Table 7:** Outcome: depression.

Study	Groups	Outcome/tool	Result: post-intervention	Result: 3-months post-intervention
Yaksi et al. (46)	1. Conventional TENS [CTENS] (*n* = 25) 2. Burst TENS [BTENS] (*n* = 25) 3. Placebo TENS [PTENS] (*n* = 23)	BDI	NA	No significant within-group or between-group changes

### 3.4 Outcome: pain

#### 3.4.1 TENS vs. active controls at post-intervention

**Figure 2:**
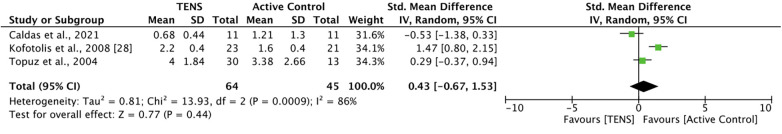
TENS vs. active controls at post-intervention (for pain).

Three studies (39, 43, 44) compared TENS with an active control. Caldas et al. (39) found that 30 min of burst TENS plus 10 min of pain education was not significantly more effective than 30 min of cryotherapy plus 10 min of pain education at reducing pain. Kofotolis et al. (43) compared 40–45 min of TENS with 30–45 min of rhythmic stabilization exercises, finding that rhythmic stabilization was significantly more effective than TENS (*p* < 0.05). Topuz et al. (44) compared 20 min of conventional TENS and 20 min of low-frequency TENS with 20 min of percutaneous neuromodulation therapy (PNT). There were no significant between-group differences in current pain, but PNT was significantly more effective than both TENS interventions at improving activity pain (*p* < 0.05). Meta-analysis revealed that active control was not significantly better than TENS at improving pain at post-intervention [SMD = 0.43, 95% CI = −0.67, 1.53] (Figure 2). The comparisons made in this analysis had a high degree of heterogeneity (*I*^2^ = 86%). For meta-analysis, the means and standards deviations of both TENS groups in Topuz et al. (44) were pooled, and “current pain” was selected as the outcome measure.

#### 3.4.2 TENS vs. active controls at ≥1-month post-intervention

Of the three studies listed above, only Kofotolis et al. (43) continued to monitor participants post-intervention. At 1-month post-intervention, the rhythmic stabilization group has significantly less pain than the TENS group (MD = 0.70, 95% CI = 0.37, 1.03). At 2-months post-intervention, the rhythmic stabilization group continued to have significantly less pain than the TENS group (MD = 0.80, 95% CI = 0.52, 1.08).

#### 3.4.3 TENS vs. passive controls at post-intervention

**Figure 3:**
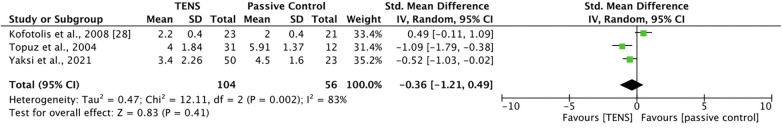
TENS vs. passive controls at post-intervention (for pain).

Three studies (43, 44, 46) compared TENS with a passive control. Kofotolis et al. (43) compared 40–45 min of TENS with 40–45 min of sham TENS. There were no significant between-group differences at post-intervention. Topuz et al. (44) compared 20 min of conventional TENS and 20 min of low-frequency TENS with 20 min of sham TENS. Both active TENS groups were significantly more effective than sham TENS at improving current pain (*p* < 0.05) and activity pain (*p* < 0.05), with no significant differences between the two active TENS groups. Yakzi et al. (46) compared the effect of 30 min of conventional TENS and burst TENS with 30 min of placebo TENS on daytime and night-time pain. At post-intervention, there was a significant difference in score change between the groups (*p* = 0.012). A Mann–Whitney *U*-test with Bonferroni correction revealed a significant difference between burst TENS and placebo TENS, with burst TENS significantly more effective (*p* = 0.007) at reducing pain. For night-time pain, there were no significant between-group differences. Additionally, Caldas et al. (39) compared TENS with sham TENS; however, the sham TENS group received the minimum therapeutic dose and was excluded from analysis. Meta-analysis revealed that TENS was not significantly better than passive control at improving pain at post-intervention [SMD = −0.36, 95% CI = −1.21, 0.49] (Figure 3). The comparisons made in this analysis had a high degree of heterogeneity (*I*^2^ = 83%). For meta-analysis, the means and standards deviations of both TENS groups in Topuz et al. (44) and Yaksi et al. (46) were pooled; additionally, “current pain” was selected as the outcome measure in Topuz et al. (44) and “daytime pain” was selected in Yaksi et al. (46).

#### 3.4.4 TENS vs. passive controls at ≥1-month post-intervention

Two studies examined the effect of TENS at 1-month post-intervention and beyond. Kotofolist et al. (43) compared TENS with sham TENS at 1 and 2-months post-intervention. There were no significant differences between the groups at either time point. Yaksi et al. (46) compared conventional and burst TENS with sham TENS at 3-months post-intervention, and reported no significant between-group differences.

#### 3.4.5 Mixed TENS vs. active controls at post-intervention

**Figure 4:**
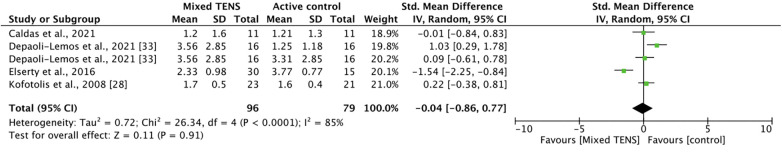
Mixed TENS vs. active controls at post-intervention (for pain).

Five comparisons across four studies studied the effect of a mixed TENS intervention vs. active control. Caldas et al. (39) compared 30 min of burst TENS/cryotherapy + 10 min of pain education with 30 min of cryotherapy + 10 min of pain education alone. Depaoli-Lemos et al. (40) compared 30 min of exercise + 20 min of TENS with 30 min of exercise alone; additionally, they compared 30 min of exercise + 20 min of TENS with 30 min of exercise + 20 min of electro-acupuncture. Elserty et al. (42) compared fixed TENS + 40 min of exercise, adjusted TENS + 40 min of exercise, and 40 min of exercise alone (for meta-analysis, the results of the TENS groups were pooled). Finally, Kofotolis et al. (43) compared rhythmic stabilization exercises + TENS with TENS alone. Meta-analysis revealed that mixed TENS was not significantly better than active control at reducing pain [SMD = −0.04, 95% CI = −0.86, 0.77] (Figure 4). The comparisons made in this analysis had a high degree of heterogeneity (*I*^2^ = 85%).

#### 3.4.6 Mixed TENS vs. active controls at 1-month post-intervention

**Figure 5:**
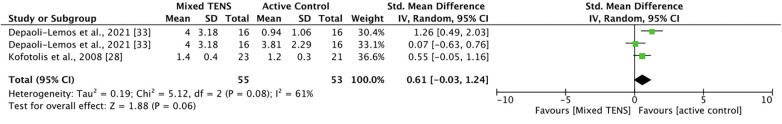
Mixed TENS vs. active controls at 1-month post-intervention (for pain).

Two studies involving three comparisons examined mixed TENS vs. active control for pain at 1-month post-intervention: two from Depali-Lemos et al. (40) and one from Kofotolis et al. (43). Meta-analysis revealed that active control was not significantly better than mixed TENS at reducing pain 1-month post intervention [SMD = 0.61, 95% CI = −0.03, 1.24] (Figure 5). The comparisons made in this analysis had a moderate degree of heterogeneity (*I*^2^ = 61%).

#### 3.4.7 Mixed TENS vs. active controls at >1-month post-intervention

Kofotolis et al. (43) compared TENS + rhythmic stabilization exercises with rhythmic stabilization exercises alone at 2-months post-intervention. There were no significant differences between the groups for pain at this timepoint.

#### 3.4.8 Mixed TENS vs. passive controls at post-intervention

Kofotolis et al. (43) compared TENS + rhythmic stabilization exercises with sham TENS. There were no significant differences between the groups for pain at this timepoint.

#### 3.4.9 Mixed TENS vs. passive controls at ≥1-month post-intervention

Kofotolis et al. (43) compared TENS + rhythmic stabilization exercises with sham TENS at 1- and 2-months post-intervention. At 1-month post-intervention, there were no significant between-group differences. However, at 2-months post-intervention, the combined group had significantly less pain than the sham TENS group (1.4 (0.4) vs. 1.9 (0.5), *p* < 0.05).

#### 3.4.10 IFC vs. active controls at post-intervention

Two studies compared the effectiveness of IFC with an active control on pain. Albornoz-Cabello et al. (37) compared IFC with a usual care intervention; the IFC group had a significantly greater reduction in pain (MD = −11.34, 95% CI = −1.77, −20.91). Lara-Palomo et al. (16) compared IFC with superficial massage; the IFC group had a significantly greater reduction in pain (MD = 1.06, 95% CI = −1.91, −0.22).

#### 3.4.11 EMS vs. active controls at post-intervention

Dimer daLuz et al. (41) compared NMES with core exercises for pain. At post-intervention, there were no significant differences between the groups.

#### 3.4.12 EMS vs. active controls at ≥1-month post-intervention

Dimer daLuz et al. (41) re-examined pain outcomes at 6-months post-intervention and reported no significant differences between the groups.

#### 3.4.13 EMS vs. passive controls at post-intervention

Batistella et al. (15) compared the effect of Russian Current with passive control (no intervention). At post-intervention, the Russian Current group had significantly less pain than the control group (median = 4, [2,4] vs. median = 4 [4,5.2], *p* = 0.0483). Pelegrini et al. (14) compared the effect of Aussie Current with passive control (no intervention). At post-intervention, there were no significant between-group differences noted on either the VAS or the McGill Pain Questionnaire. However, the Aussie Current group had significantly greater pain at baseline (*p* = 0.0063, ES = 1.16).

#### 3.4.14 EMS vs. passive controls at 1-month post-intervention

At 1-month post-intervention, Batistella et al. (15) reported no significant differences in pain between the Russian Current and control groups, while Pelegrini et al. (14) found that the Aussie Current group had significantly less pain than the control group as reported on the VAS (32.60 (26.65) vs. 55.00 (9.05), *p* = 0.0147), but not the McGill Pain Questionnaire.

#### 3.4.15 Mixed EMS vs. active controls at post-intervention

Three studies investigated the effect of mixed EMS vs. active control. Due to reporting differences, only two studies were eligible for meta-analysis, and therefore none was conducted. Alrwaily et al. (38) compared the effect of NMES + stabilization exercises with stabilization exercises alone and reported no significant between-group differences for pain at post-intervention. Dimer daLuz et al. (41) compared NMES + core exercises with core exercises alone. At post-intervention, there were no significant differences between the groups. Finally, Weissenfels et al. (45) compared WB-EMS with conventional back strengthening exercises. At post-intervention, there were no significant between-group differences in change in pain from baseline (*p* = 0.160).

#### 3.4.16 Mixed EMS vs. active controls at ≥1-month post-intervention

Dimer daLuz et al. (41) re-examined pain outcomes at 6-months post-intervention and reported no significant differences between the groups.

#### 3.4.17 Mixed EMS vs. passive controls at post-intervention

Weissenfels et al. (13) compared the effect of WB-EMS with passive control (no intervention). At post-intervention, the WB-EMS group had achieved a significantly greater reduction in pain from baseline compared to the control group (MD = 0.67, 95% CI = 0.18, 1.21, *p* = 0.028).

### 3.5 Outcome: disability

#### 3.5.1 TENS vs. active controls at post-intervention

**Figure 6:**
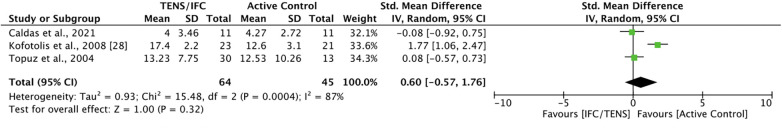
TENS vs. active controls post-intervention (for disability).

Three studies compared TENS with active controls for disability. Caldas et al. (39) did not find significant difference between TENS and cryotherapy at post-intervention. Kofotolis et al. (43) reported that rhythmic stabilization exercises were significantly more effective than TENS at improving disability (MD = 4.80, 95% CI = 3.20, 6.40). Topuz et al. (44) found no significant differences on both the ODI and LBPOS between PNT and either conventional or low-frequency TENS at post-intervention. Meta-analysis revealed no significant difference between TENS and active control [SMD = 0.60, 95% CI = −0.57, 1.76] (Figure 6). The comparisons in this analysis had a high degree of heterogeneity (*I*^2^ = 87%).

#### 3.5.2 TENS vs. active controls at ≥1-month post-intervention

Only one study examined the effect of TENS at 1-month post-intervention and beyond. At 1-month post-intervention, Kofotolis et al. (43) reported that rhythmic stabilization exercises were significantly more effective than TENS at improving disability (MD = 7.10, 95% CI = 6.28, 7.92). At 2-months post-intervention, Kofotolis et al. (43) found that rhythmic stabilization exercises continued to be significantly more effective than TENS at improving disability (MD = 6.40, 95% CI = 4.85, 7.95).

#### 3.5.3 TENS vs. passive controls at post-intervention

Two studies compared the effect of TENS with passive controls on disability. Kofotolis et al. (43) reported that TENS was not significantly more effective than sham TENS at improving disability. Topuz et al. (44) reported that both conventional TENS and low-frequency TENS were more effective than sham TENS at improving disability (using the ODI) at post-intervention (*p* < 0.05). After pooling the two TENS groups, the combined group continued to demonstrate greater effectiveness than sham TENS (MD = −5.14, 95% CI = −9.18, −1.10). However, when reporting with the LBPOS, while both conventional and low-frequency TENS were more effective than sham TENS at improving function at post-intervention (both *p* < 0.05), this effect was not significant when the groups were pooled (MD = 4.75, 95% CI = −2.95, 12.45).

#### 3.5.4 TENS vs. passive controls at ≥1-month post-intervention

Two studies examined the effect of TENS at 1-month post-intervention and beyond. Kofotolis et al. (43) reported no difference in disability between TENS and sham TENS at 1-month and 2-months post-intervention. Yaksi et al. (46) reported no significant differences in disability between the conventional TENS, burst TENS, and placebo TENS groups at 3-months post-intervention.

#### 3.5.5 Mixed TENS vs. active controls at post-intervention

**Figure 7:**
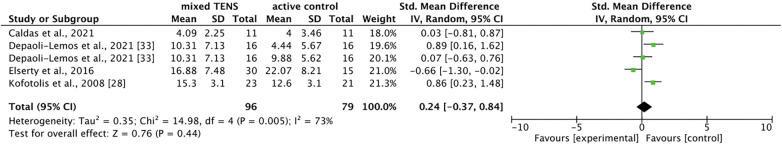
Mixed TENS vs. active controls post-intervention (for disability).

Five comparisons across four studies (39, 40, 42, 43) compared mixed TENS with active control for disability. Meta-analysis revealed no significant difference between mixed TENS and active control [SMD = 0.24, 95% CI = −0.37, 0.84] (Figure 7). The comparisons made in this analysis had a moderate degree of heterogeneity (*I*^2^ = 73%).

#### 3.5.6 Mixed TENS vs. active controls at ≥1-month post-intervention

**Figure 8:**
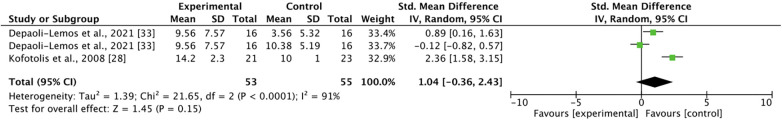
Mixed TENS vs. active controls at ≥1-month post-intervention (for disability).

Two studies involving three comparisons examined mixed TENS vs. active control for pain at 1-month post-intervention: two from Depali-Lemos et al. (40) and one from Kofotolis et al. (43) Met-analysis revealed no significant differences between mixed TENS and active control [SMD = 1.04, 95% CI = −0.36, 2.43] (Figure 8). The comparisons in this analysis had a high degree of heterogeneity (*I*^2^ = 91%). At 2-months post-intervention, Kofotolis et al. (43) reported that rhythmic exercise continued to be significantly more effective than combined TENS + rhythmic stabilization exercises at reducing disability (9.9 (0.8) vs. 13.7 (1.9), *p* < 0.05).

#### 3.5.7 Mixed TENS vs. passive controls at post-intervention

Kofotolis et al. (43) investigated the effect of TENS + rhythmic stabilization exercises vs. sham TENS on disability. At post-intervention, there were no between-group differences.

#### 3.5.8 Mixed TENS vs. passive controls at ≥1-month post-intervention

At 1-month post-intervention, Kofotolis et al. (43) found that the mixed TENS group had significantly less disability than the sham TENS group (14.2 (2.3) vs. 16.5 (2.0), *p* < 0.05). At 2-months post-intervention, this finding continued to hold (13.7 (1.9) vs. 15*.*8 (1.9), *p* < 0.05).

#### 3.5.9 IFC vs. active controls at post-intervention

Both Albornoz-Cabello et al. (37) and Lara-Palomo et al. (16) investigated the effect of their intervention on disability. Albornoz-Cabello et al. (37) reported that IFC was significantly more effective than usual care (MD = −13.38, 95% CI = −21.78, −4.97), while Lara-Palomo et al. (16) found that IFC was more effective than superficial massage as reported with both the ODI (MD = −5.20, 95% CI = −10.82, 0.42) and the RMDQ (MD = −3.01, 95% CI = −4.53, −1.47).

#### 3.5.10 EMS vs. active controls at post-intervention

Dimer daLuz et al. (41) reported no significant differences between the NMES and core-exercise groups on both the ODI and RMDQ.

#### 3.5.11 EMS vs. active controls at ≥1-month post-intervention

At 6-months post-intervention, Dimer daLuz et al. (41) continued to find no significant differences between the NMES and core-exercise groups.

#### 3.5.12 EMS vs. passive controls at post-intervention

Neither Batistella et al. (15) nor Pelegrini et al. (14) reported significant differences between their active intervention (NMES and Aussie Current, respectively) and passive control for disability at post-intervention.

#### 3.5.13 EMS vs. passive controls at 1-month post-intervention

Neither Batistella et al. (15) nor Pelegrini et al. (14) reported significant differences between their active intervention (NMES and Aussie Current, respectively) and passive control for disability at 1-month post-intervention.

#### 3.5.14 Mixed EMS vs. active controls at post-intervention

Both Alrwaily et al. (38) and Dimer daLuz et al. (41) investigated the effect of their intervention on disability. Alrwaily et al. (38) reported no significant between-group differences. Dimer daLuz et al. (41) found that the mixed EMS group had significantly less disability than the core exercise group on the ODI [1.2 (0.91) vs. 5.17 (3.19), *p* < 0.05], but not on the RMDQ [0.5 (0.85) vs. 2.6 (3.06), *p* > 0.05].

#### 3.5.15 Mixed EMS vs. active controls at ≥1-month post-intervention

Dimer daLuz et al. (41) re-examined disability outcomes at 6-months post-intervention and reported no significant differences between the groups on both the ODI and RMDQ.

### 3.6 Outcome: quality of life

#### 3.6.1 TENS vs. active controls at post-intervention

Two studies compared the effect of TENS with active controls on quality of life. Caldas et al. (39) reported no difference between TENS and cryotherapy on any of the SF-36 domains at post-intervention. Topuz et al. (44) found that PNT was significantly more effective than conventional TENS at improving the *emotional role limitations* domain of the SF-36 (*p* < 0.05), significantly more effective than conventional TENS at improving the *vitality* domain of the SF-36 (*p* < 0.05), and significantly more effective than both conventional and low-frequency TENS at improving the *general health perception* domain of the SF-36 (both *p* < 0.05) at post-intervention.

#### 3.6.2 TENS vs. passive controls at post-intervention

One eligible study compared the effect of TENS with sham TENS on quality of life. Topuz et al. (44) reported that both conventional and low-frequency TENS were significantly more effective than sham TENS at improving the following quality-of-life domains: *physical functioning, social functioning, physical role limitation, general mental, bodily pain,* and *general health perception* (all *p* < 0.05). Low-frequency TENS was significantly more effective than sham TENS at improving the *emotional role limitations* domain (*p* < 0.05), but sham TENS was significantly more effective than conventional TENS (*p* < 0.05).

#### 3.6.3 IFC vs. active controls at post-intervention

Lara-Palomo et al. (16) investigated the effect of IFC on quality-of-life. IFC was more effective than superficial massage in improving quality of life in the following domains: *physical function* (MD = 5.57, 95% CI = −2.27, 13.41), *physical role* (MD = 7.02, 95% CI = 1.05, 12.98), *body pain* (MD = 4.27, 95% CI = −0.28, 9.71).

### 3.7 Outcome: fear-avoidance beliefs

#### 3.7.1 Mixed EMS vs. active controls at post-intervention

Alrwaily et al. (38) considered the effect of their intervention on work- and physical activity-related fear avoidance beliefs. At post-intervention, there were no significant between-group differences for either work- or physical activity-related fear avoidance beliefs.

### 3.8 Outcome: depression

#### 3.8.1 TENS vs. passive control at 3-months post-intervention

Yaksi et al. (46) reported no significant within- or between-group differences in depression at 3-months post-intervention.

## 4 Discussion

To our knowledge, this is the first meta-analysis to evaluate the effect of a variety of transcutaneous electrotherapies on several PROMs for CLBP. Previous systematic reviews reported on the effect of TENS for pain [Van Tulder et al. (20); Flowerdew et al. (21); Milne et al. (22); Khadlikar et al. (23); Poitras and Brosseau (24); Jauregui et al. (25); Wu et al. (26)) and TENS for disability/function (Milne et al. (22); Poitras and Brosseau (24); Khadilkar et al. (23); Wu et al. (26)] for CLBP patients. The most recent systematic reviews reported a modest but positive effect on pain and function under certain conditions. Jauregui et al. (2016) found significant weighted mean differences in pain intensity in patients treated with TENS for less than, but not more than, 5 weeks (25) Wu et al. (2018) found that TENS improves functional disability when follow-up is within 6-weeks of treatment, compared to controls (26) To date, no systematic reviews had examined the effect of TENS on pain catastrophizing or fear-avoidance beliefs in CLBP patients. Additionally, no systematic reviews had been able to report the effect of EMS on PROMs, while a single review reported on the effect of IFC for pain in a variety of musculoskeletal conditions including CLBP (17). An additional novelty of this meta-analysis is that stand-alone and mixed interventions were examined separately at multiple time-points.

The effect of transcutaneous electrotherapies on pain, compared to active and passive controls, appears to be intervention-dependant. The results of the meta-analyses for TENS on pain revealed no significant differences between TENS and either active or passive controls at post-intervention. This finding is similar to Wu et al. (26), who reported no significant differences between TENS and passive control for pain [SMD: −0.20, 95% CI: −0.58, 0.18, *p* = 0.293]. Wu et al. (26) additionally found that neural stimulation therapy (NST) was more effective than TENS at reducing pain [SMD: 0.68, 95% CI: 0.15, 1.57, *p* = 0.017]. Although our meta-analysis did not reveal a significant difference between TENS and active control, our definition of active control was very broad, which may explain the discrepancy with Wu et al. (2018). A single study (43) re-assessed pain at 1- and 2-months post-intervention and reported that the active control group had less pain than the TENS group at both time points. Similarly, meta-analyses for the effect of mixed TENS revealed no significant differences between active control at post-intervention and 1-month post-intervention. There were also no reported differences between mixed TENS and active control at 2-months post-intervention (43) or between mixed TENS and passive control and post intervention (43) and 1-month post intervention (43) However, one study reported that the mixed TENS group had significantly less pain than passive controls at 2-months post-intervention (43) On the other hand, the two studies that investigated IFC (16, 37) both reported that it was more effective than active control at reducing pain at post-intervention. Amongst EMS studies, stand-alone EMS was not superior to active control at post-intervention (41) and 6-months post-intervention (41), but it was more effective than passive control at post-intervention (15) and 1-month post-intervention (14). Similarly, mixed EMS was similar to active control at post-intervention (38, 41, 45) and 6-months post-intervention (41), but was more effective than passive control at post-intervention (13).

The results of this synthesis suggest that both IFC and EMS may be more suitable interventions for reducing pain at post-intervention than TENS, since they performed better than TENS vs. active controls (the IFC studies) and passive controls (the EMS studies). Nevertheless, this finding should be interpreted with caution, given that no meta-analysis for IFC or EMS was performed. Previously, a 2020 overview of Cochrane Reviews (47) examined eight Cochrane Reviews of TENS for chronic pain, involving 51 RCTs and spanning 2,895 participants, but was unable to conclude with confidence whether TENS was beneficial or safe for pain control. Similarly, a recent comparative review of electrical stimulation (ES) devices found that the efficacy of TENS for pain was low-to-insignificant (48). In our systematic review, an analysis of within-group changes for all active transcutaneous electrotherapy interventions suggests that TENS, IFC, and EMS are generally effective at reducing pain at post-intervention: 15 interventions resulted in a significant reduction in pain (6 TENS, 2 IFC, 7 EMS), while 2 did not (2 TENS). Additionally, a recent meta-analysis of TENS for chronic pain (the meta-TENS study) (49) reported that TENS was more effective than placebo for pain relief during or immediately post-application [SMD: −0.96, 95% CI: −1.14, 0.78; moderate-certainty evidence] and more effective than pharmacological and non-pharmacological treatment for pain relief during or immediately post-application [SMD: −0.72, 95% CI: −0.95, −0.50; low-certainty evidence]. Clinicians may want to prioritize the use of IFC or EMS as part of a longer-term treatment plan, especially since recent systematic reviews reported that EMS is effective at improving trunk muscle strength (30) and endurance (30, 31) in CLBP patients, but TENS may be suitable for short-term pain relief within a multi-modal treatment plan.

The efficacy of transcutaneous electrotherapies at reducing disability was mixed. For TENS interventions, one study found that active control was more effective at post-intervention and ≥1-month post-intervention (43), while another reported no significant differences at post-intervention (44). Some studies found that TENS was more effective than passive control (44), while others found no difference (43, 46). Mixed TENS was more effective than passive controls at ≥1-month post-intervention (43), but not more effective at earlier time points (43) or compared to active controls at any time point (42, 43) Additionally, there were no significant differences in effect on disability for any of the EMS interventions at any time point, with the exception of Dimer da Luz et al. (41), who reported that mixed EMS was significantly more effective than active control at post-intervention. On the other hand, both IFC studies (16, 37) reported that it was significantly more effective than active control at reducing disability at post-intervention. An analysis of within-group changes for active transcutaneous electrotherapy interventions suggests an overall benefit for disability at post-intervention: 8 interventions resulted in a significant reduction in disability (2 TENS, 2 IFC, 4 EMS), while 3 did not (2 TENS, 1 EMS). Previous systematic reviews reported no overall difference in disability between TENS and passive controls (22, 23, 26), although one found that TENS was superior to passive control when follow-up was less than 6-weeks [SMD: −1.24, 95% CI: −1.83, −0.65, *p* < 0.001] (26) Additionally, Wu et al. (26) reported no difference in disability between TENS and NST.

### 4.1 Limitations

Due to the small number of studies that investigated quality-of-life, fear-avoidance belief, and depression outcomes, no meta-analyses were performed for any of these outcome measures. We were also unable to find eligible studies that investigated pain catastrophizing. Therefore, we could not draw meaningful conclusions about the effectiveness of transcutaneous electrotherapies on these outcome measures. Another limitation of this systematic review is a lack of consistency with the included studies' definition of CLBP and inclusion criteria. Ten studies defined CLBP as low back pain of at least 3 months duration (14–16, 37–42, 44), two defined it as low back pain experienced on ≥ 50% of days in the previous 3 months (13, 45), one defined it as mechanical back pain of at least 3 months duration from degenerative disc disease or disc herniation without radicular compression (46), and one defined it as “chronic”—without specifying a baseline for symptom duration—whilst noting that participants were recruited from a pool of individuals who had LBP for at least 6 months (43). Studies also differed with respect to baseline levels of pain or disability. Five studies required participants to have at least a moderate amount of pain or disability at baseline (16, 37–39, 41) and the remaining nine did not (13–15, 40, 42–46). Requiring study participants to have at least a moderate degree of pain or disability allows for minimum important changes (MIC) to be detected. This value has been reported for several LBP questionnaires that our included studies used: the Visual Analogue Scale (MIC = 15), Numerical Pain Rating Scale (MIC = 2), the Oswestry Disability Index (MIC = 20%), and the Roland Morris Disability Questionnaire (MIC = 5) (50). Of the ten studies that used the ODI, participants in at least one study arm of four studies (14, 15, 43, 44) had baseline ODI scores of <20%, which may have limited improvements in disability.

A significant limitation to the generalizability of our findings was the high level of heterogeneity (> 80%) that was observed in five out the seven meta-analyses we conducted. This heterogeneity is likely due to between-study differences in active controls and sample characteristics. For example, in the comparison between TENS and passive controls for immediate pain relief, the one study (43) that did not report a significant between-group difference in pain had a sample comprised entirely of women, while the other two (44, 46) were comprised of sexes. While it is possible that the outcomes observed reflect sex-based differences in response to TENS, meta-analysis with a random-effects model—which we used—tends to give each sample a similar weight when calculating the mean difference (51). The broad nature of our study question—how effective are common transcutaneous electrotherapies for CLBP compared with controls?—meant that we were likely to include studies with parameters that differed in significant ways, leading to limits to the interoperability of our results, especially when they were not significant.

One feature of this systematic review was our stringent exclusion criteria. We only included studies with at least 5 participants per study arm, at least 8 treatments per group, with participants no older than 70 years without radicular CLBP or spinal abnormalities, and where the method of electrotherapy application (including stimulation intensity) was precisely described. As a result, we excluded several studies that were in included in previous systematic reviews (23, 24, 26) to increase the specificity of our findings. Although our TENS results were not significantly from those previously reported with respect to pain intensity and disability, our methodology provides insight into the effect of longer-term treatment with transcutaneous electrotherapies, which has not been the focus of previous systematic reviews.

Lastly, all our included studies had at least a moderate risk of bias, in large part because of the lack of availability of study protocols, which elevated the risk of reporting bias. Future RCTs can minimize the risk of bias by publishing the study protocol alongside the final report.

### 4.2 Conclusion

In sum, there is *moderate* evidence that TENS is similar to both active and passive controls for improving pain and disability in CLBP patients. There is *limited* evidence that IFC is superior to active controls for improving pain and disability. There is *limited* evidence that EMS is superior to passive but not active controls for improving pain, and similar to all controls for improving disability. Finally, there is *inconclusive* evidence regarding the effect of transcutaneous electrotherapies on quality-of-life and fear avoidance beliefs due both to the limited number of studies investigating these outcomes and, for quality-of-life, the high number of domains listed on the SF-36. Future transcutaneous electrotherapy investigations may want to prioritize EMS and medium-frequency interventions, since these are understudied compared to TENS, appear to be more promising for PROMs, and can also lead to functional improvements in strength and endurance (30, 31). Additionally, research into promising but less-studied forms of transcutaneous electrotherapy is justified; for example, into H-wave Device Stimulation, which was recently reported to improve pain, sleep and work performance in CLBP patients (52). Finally, the impact of all transcutaneous electrotherapy interventions on psychological mediators such as kinesiophobia and pain catastrophizing warrants further study.

## Data Availability

The original contributions presented in the study are included in the article/Supplementary Material, further inquiries can be directed to the corresponding author.

## Footnotes

^1^ http://who.int/ictrp/en/

^2^ https://clinicaltrials.gov

^3^ https://sr-accelerator.com

^4^https://www.statstodo.com/CombineMeansSDs.php)

## Appendix 1 Sample Search Strategy

(PubMed)

#1 Search: “Low Back Pain” [Mesh] OR “Back Pain” [Mesh:NoExp] OR “Hernia” [Mesh:NoExp] OR “Intervertebral Disc Displacement” [Mesh] OR “Intervertebral Disc Degeneration”[Mesh] OR “lumbosacral region” [Mesh] OR “Low back pain” OR “lumbago” OR “disc herniation” OR “intervertebral disc herniation” OR “disc degeneration” OR “degenerative disc” OR “lower back pain” OR “back ache” OR “backache” OR “backaches” OR “sacral pain” OR “lumbar pain” OR “lumbosacral pain” OR “LBP”

#2 Search: “Transcutaneous Electric Nerve Stimulation” [Mesh] OR “Electric Stimulation Therapy” [Mesh:NoExp] OR “Electric Stimulation” [Mesh:NoExp] OR “electric stimulation” OR “electrical stimulation” OR “electrostimulation” OR “TENS” OR “nerve stimulation” OR “electrotherapy” OR “interferential current” OR “electroanalgesia” OR “IFC” OR “neuromuscular electrical stimulation therapy” OR “NMES” OR “electromyostimulation” OR “EMS” OR “ES” OR “functional electrical stimulation” OR “FES” OR “Whole body electromyostimulation” OR “whole-body electromyostimulation” OR “WB-EMS”

#3 Search: (Animal[Mesh] NOT Human[Mesh])

#4 Search: #1 AND #2

#5: #4 NOT #3

(Embase)

#1 Search: (low back pain or low back pain).mp

#2 Search: (transcutaneous electric* nerve stimulation or transcutaneous electrical nerve stimulation).mp

#3 Search: (neuromuscular electric* stimulation or neuromuscular electric* nerve stimulation or neuromuscular electrical stimulation).mp

#4 Search: electrotherapy/or high frequency electrotherapy/or low frequency electrotherapy/or electrotherapy.mp

#5 Search: (interferential current or IFC or functional electric* stim* or FES or whole-body electromyostim* OR WB-EMS).mp

#6 Search: 2 or 3 or 4 or 5

#7 Search: 1 and 6.
